# Advancing the Development of Subcutaneous Glucose Biosensors: Cargo‐Carrying Adhesive Biosensor Systems (CABs)

**DOI:** 10.1002/mabi.202500445

**Published:** 2025-10-12

**Authors:** Carolina I. Martinez, Theodore S. Ferrell, Varshitha M. Krishnan, Melissa A. Grunlan

**Affiliations:** ^1^ Department of Chemistry Texas A&M University College Station Texas USA; ^2^ Department of Biomedical Engineering Texas A&M University College Station Texas USA; ^3^ Department of Biomedical Engineering Department of Materials Science & Engineering Department of Chemistry Center for Remote Health Technologies Systems Texas A&M University College Station Texas USA

**Keywords:** biosensors, double network, electrostatic adhesion, hydrogels, thermoresponsive

## Abstract

The development of an injectable, subcutaneous glucose biosensor may be advanced by utilizing optical glucose sensing assays. However, this requires a strategy to effectively house small‐sized assay molecules. Herein, “CABs” or cargo‐carrying adhesive biosensors were constructed via the electrostatic adhesion of a hollow rod membrane (whose cavity could store a liquid optical‐assay) and hydrogel caps. For the CAB ‘wall’, the hollow rod leveraged a comb double network (DN) design previously shown to limit biofouling and reduce mesh size. To regulate the mesh size, poly(AMPS)‐methacrylate (PAMPS_n_‐MA) comb macromers were incorporated into the 1st network. To improve electrostatic adhesivity, anionic 2‐acrylamido‐2‐methylpropane sulfonic acid (AMPS) was incorporated into the second network of the DN hydrogels at varying concentrations. For the CAB ‘cap’ with a cationic surface, a semi‐interpenetrating polymer network was formed, comprised of crosslinked polyampholyte and non‐crosslinked cationic polyelectrolyte. A CAB was constructed with a CAB wall based on a DN hydrogel composition shown to exhibit the requisite thermosensitivity, mechanical robustness, glucose diffusivity, low mesh size (4 nm <* ξ* < 7 nm), and adhesivity to the CAB cap. Using FITC‐dextran solutions, the CAB was shown to retain ∼90% of molecules of low hydrodynamic diameters (*D_h_ ∼ *7 nm and *D_h_* *∼ *10 nm).

## Graphical abstract

A “CAB” or “cargo‐carrying adhesive biosensor” was constructed by leveraging electrostatic adhesion between an anionic hollow rod membrane (whose cavity could store a liquid optical‐assay) and cationic hydrogel caps. A final CAB was constructed from the optimal CAB wall candidate and CAB cap, and successfully retained small molecules (hydrodynamic diameter: *∼*7 and *∼*10 nm) within the central cavity.

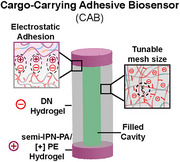

## 1 Introduction

The most prevalent form of diabetes management, self‐monitoring of blood glucose via finger‐prick testing, increases the occurrence of hyper‐ and hypoglycemic events due to its intermittent nature [1, 2, 3]. In response, continuous glucose monitors (CGMs) have emerged as improved glucose management systems by providing real‐time, continuous glycemic values [2, 4, 5, 6]. Several on‐the‐market CGMs utilize disposable, transdermal electroenzymatic probes for glucose concentration detection in the interstitial fluid (ISF) [5]. However, in addition to probe insertion site irritation and the requirement of a skin‐affixed transmitter, transdermal probe biofouling (i.e., protein and cellular accumulation) leads to compromised glucose diffusion. As a result, these transcutaneous CGMs have limited lifetimes ranging (∼7–14 days) [7, 8]. Subcutaneous (‘sub‐Q’) dwelling CGMs, contingent on the ability to mitigate biofouling to reduce replacement frequency, may result in improved user comfort [9, 10].

Optical glucose sensing technologies hold promise in the development of sub‐Q CGMs. The Eversense 365 CGM is the only such CGM currently available [9, 10, 11]. A lifetime of up to 365 days is potentially achieved through the slow release of dexamethasone acetate, an anti‐inflammatory corticosteroid, to limit surface biofouling [9]. However, its size (∼0.17“ × ∼0.75”, *d × l*) requires incision for implantation, and a bulky skin‐affixed transmitter is also needed [12]. A long‐term functioning, sub‐Q CGM whose size permits injectability, and that eliminates the use of bulky transmitters would be a superior alternative. Emerging liquid optical glucose‐sensing assays represent a promising approach [13, 14, 15, 16, 17]. Optical glucose sensors employ numerous glucose‐sensing methods [18, 19, 20, 21], including those based on fluorescence [13, 14, 17, 19, 22], near‐infrared (NIR) [17, 23], Raman [24, 25], phosphorescence [26], and photoacoustic [27]. Because red and NIR light penetrate skin more deeply than UV or blue light, a fluorescence‐based assay that employs red and NIR probes would be advantageous [17, 28]. This could enable a smaller biosensor size for sub‐Q injectability, such as at the wrist, and utility among populations of varying skin tones and body mass indices. This type of biosensor could be conveniently probed with a watch‐like device that employs an external LED light source (fluorophore excitation) and photodiode (signal measurement) [18, 29, 30]. The utility of optical glucose sensing assays requires that they are ‘packaged’ into a carrier that adequately controls biofouling, and also simultaneously achieves assay molecule retention and glucose diffusion. Based on their hydrated nature and semi‐permeable diffusivity, hydrogel membranes represent a promising carrier for sub‐Q optical‐based glucose biosensors.

A hydrogel carrier must mitigate biosensor biofouling by minimizing the foreign body reaction (FBR), namely the accumulation of proteins and various cell types (e.g., neutrophils, macrophages, etc.) that lead to the eventual formation of a glucose‐diffusion limiting fibrous capsule. Numerous methods have been explored to combat biofouling on implanted glucose biosensors, predominantly employing passive anti‐biofouling mechanisms wherein fouling events are reduced through inert surfaces such as hydrogel membranes, as well as other approaches such as drug‐elution, and biomimetic coatings [12, 31, 32] or via the release of bioactive agents (e.g., dexamethasone acetate, nitric oxide) [33, 34, 35, 36]. In contrast, we have reported an ‘active’ anti‐biofouling strategy using a thermoresponsive hydrogel membrane. This membrane (‘*G_1_* ′ – first generation) was formed as an electrostatic, double network (DN) hydrogel based on thermoresponsive poly‐*N*‐isopropylacrylamide (PNIPAAm) [37, 38, 39]. It consisted of a tightly crosslinked first network based on NIPAAm and anionic [−] acrylamido‐2‐methylpropanesulfonic acid (AMPS) [75:25 wt.% ratio of NIPAAm: [−] AMPS], and a loosely crosslinked second network based on NIPAAm and *N*‐vinylpyrrolidone (NVP). NVP was used to precisely tune the volume phase transition temperature (VPTT) [*T*_onset_ ∼ 37°C and *T*_max_ ∼ 41°C]. In this way, the membrane exists in a predominantly swollen state, but would undergo cyclical deswelling/reswelling in response to temperature fluctuations experienced in the wrist sub‐Q tissue. Cyclical, temperature‐driven deswelling/reswelling was shown to trigger the detachment of adhered cells in vitro [38, 39, 40]. When injected into the sub‐Q tissue of rats, *G_1_* hydrogel rods (∼1 mm × ∼5 mm, *d* × *l*) resulted in an exceptionally thin fibrous capsule (∼20–25 µm) over 90 days versus a non‐thermoresponsive poly(ethylene gycol)‐diacryalate (PEG‐DA, 10% w/v, 3.4k g/mol) (‘*PEG*’) hydrogel control of generally accepted biocompatibility [37]. Additionally, the asymmetrically crosslinked DN design and dynamic bonding imparted by electrostatic repulsive interactions (via [−] AMPS) and hydrophobic associations (via NIPAAm) resulted in mechanical robustness, including greater compressive modulus (*E_c_ *∼ 0.5 MPa) and compressive strength (*σ_c_* ∼ 3.34 MPa) versus the PEG hydrogel (*E_c_ ∼ 0.22 *MPa, *σ_c_ *∼ 0.13 MPa) [37].

Membrane diffusivity is an integral aspect to its utility to form a sub‐Q glucose biosensor with optical glucose‐sensing assays. However, retention of small‐sized assay molecules within a hydrated hydrogel membrane is challenging. Conventional hydrated hydrogels, owing to their mesh size (∼5–100 nm) [41, 42, 43, 44, 45], are unable to retain molecules with small hydrodynamic diameters (*D_h_*) (*D_h_* < 5 nm). This includes emerging optical glucose sensing assay molecules such as fluorescently labeled glucose/galactose‐binding protein that changes conformation when bound (*D_h_*: 5.4 nm) and unbound (*D_h_*: 6.4) nm to glucose [46]. For this reason, it is imperative to develop hydrogels of sufficiently low mesh size to retain small assay molecules while still permitting adequate diffusion of glucose (*D_h_ *∼ 0.8 nm). Reduction of hydrogel mesh size is typically achieved by increasing the polymer concentration and/or crosslink density (e.g., decreased polymer molecular weight, and/or increased concentration of crosslinker), but may have adverse impacts on key material properties (e.g., reduced hydration), resulting in diminished biocompatibility [41, 47, 48]. To reduce the mesh size of *G_1_*_,_ we prepared *G_2_* by incorporating a comb macromer, anionic poly(AMPS)‐methacrylate ([−] PAMPS_10_‐MA) into the first network, but maintained key material properties (e.g., thermosensitivity and hydration) [49]. If formed as a hollow rod, such a DN hydrogel could ultimately house a liquid optical glucose‐sensing assay within the central cavity, but would additionally require effective sealing of the cavity ends.

**FIGURE 1:**
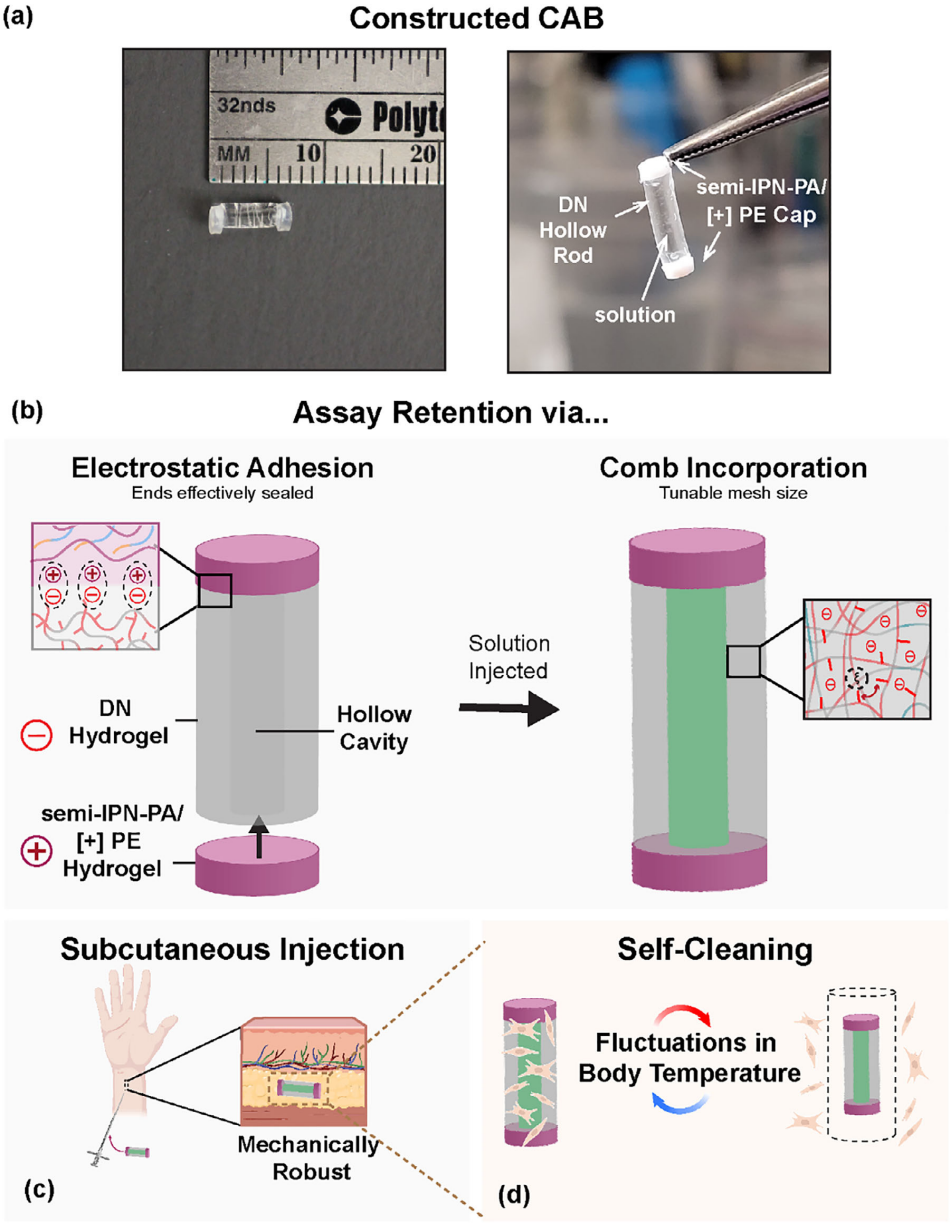
(a) (left) Photo of a constructed cargo‐carrying adhesive biosensor (CAB), and (right) ability to retain solutions within the central cavity. (b) Electrostatic adhesion between CAB walls (i.e., DN hydrogel with an anionic [−] surface) and CAB caps (i.e., polyampholyte semi‐interpenetrating polymer network (sIPN) with a cationic [+], non‐crosslinked polyelectrolyte – semi‐IPN‐PA/[+]PE). This design supports (c) mechanical robustness before and after sub‐Q injection, and (d) reduced biofouling via thermally‐driving self‐cleaning (i.e., deswelling/reswelling).

Herein, we sought to develop a new hydrogel carrier system – comprised of a hollow rod and caps that adhere to each other – that could be readily assembled to house emerging optical assays and thereby contribute to the development of a sub‐Q glucose biosensor with long‐term efficacy. Adhesive hydrogels for in vivo use are often limited by low modulus, cytotoxicity, and interfacial bound water that limits adhesive interactions in wet environments [45, 50, 51, 52]. However, one such method to impart adhesivity onto hydrogels is through the incorporation of ionic moieties [50, 53, 54, 55]. Herein, we report the development of a “CAB” or cargo‐carrying adhesive biosensor comprised of a “self‐cleaning” DN hollow rod hydrogel ‘walls’ that is sealed with hydrogel ‘caps’ (Figure 1). We sought to facilitate their electrostatic adhesivity by imparting an anionic and cationic surface to the walls and caps, respectively. Thus, CAB wall candidates were prepared similarly to *G_1_* and *G_2_*, but with [−] AMPS introduced into the second network at tunable wt.% ratios of NVP to [−] AMPS (Table 1). Additionally, to control and reduce the mesh size, [‐] PAMPS_n_‐MA combs of various lengths (*n *= 10 for *G_3_‐series*, and *n *= 20 for *G_4_‐series*) were incorporated into the first network. The CAB cap was formed from a semi‐interpenetrating polymer network comprised of crosslinked polyampholyte and non‐crosslinked cationic polyelectrolyte (semi‐I*PN‐PA/[+]PE*). The DN hydrogel CAB wall candidates were evaluated in terms of their thermosensitivity, hydration, mechanical properties, mesh size, glucose diffusion, and adhesivity to the CAB cap hydrogel. Last, CABs were assembled and their ability to retain small molecules in the central cavity examined.

**TABLE 1:** DN hydrogel “CAB Wall” candidate compositions.

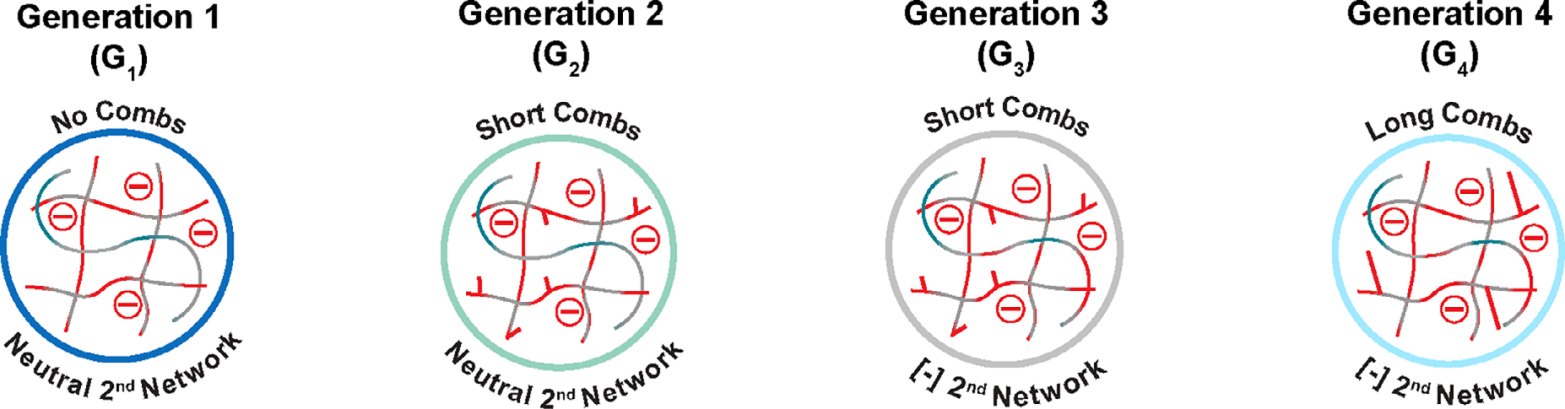								
		First Network^a^ (1.11 m total monomer)			Second Network^b^			
		85 mol %	15 mol %			5% w/v		
		NIPAAm	AMPS	PAMPS_n_‐MA	NIPAAm	NVP	AMPS	%
	Notation	(mol%)	(mol%)	(mol%)	(m)	(w/v%)	(w/v%)	AMPS^c^ ^)^
**Generation 1**	**G_1_**	85	15	0	2.5	5	0	0
**Generation 2** (PAMPS_n_‐MA, *n* = 10)	**G_2_**	85	11.25	3.75	2.5	5	0	0
**Generation 3**	**G_3_**	85	9.75	5.25	2.5	5	0	0
(PAMPS_n_‐MA, *n* = 10)	**G_3_‐AMPS_50_**	85	9.75	5.25	2.5	2.5	2.5	50
	**G_3_‐AMPS_60_**	85	9.75	5.25	2.5	2	3	60
	**G_3_‐AMPS_70_**	85	9.75	5.25	2.5	1.5	3.5	70
	**G_3_‐AMPS_80_**	85	9.75	5.25	2.5	1	4	80
	**G_3_‐AMPS_90_**	85	9.75	5.25	2.5	0.5	4.5	90
	**G_3_‐AMPS_100_**	85	9.75	5.25	2.5	0	5	100
**Generation 4**	**G_4_**	85	12.375	2.625	2.5	5	0	0
(PAMPS_n_‐MA*, n* = 20)	**G_4_‐AMPS_60_**	85	12.375	2.625	2.5	2	3	60
	**G_4_‐AMPS_70_**	85	12.375	2.625	2.5	1.5	3.5	70
	**G_4_‐AMPS_80_**	85	12.375	2.625	2.5	1	4	80

^a^ 3.3 mol% BIS crosslinker w.r.t total monomer concentration, 4.6 mol% Irgacure 2959 photoinitiator w.r.t total monomer concentration.

^b^ 0.15 mol% BIS crosslinker w.r.t NIPAAm, 2 mol% Irgacure 2959 photoinitiator w.r.t total monomer concentration.

^c^ $\%\;\mathrm{AMPS}=\frac{\mathrm{AMPS}\;(w/v\;\%)}{\mathrm{NVP}\;(w/v\;\%)+\;\mathrm{AMPS}\;(w/v\;\%)}\times 100.$

## 2 Results and Discussion

### 2.1 Hydrogel CAB Wall – Fabrication

To enable the realization of a sub‐Q glucose biosensor capable of housing small molecule optical glucose sensing assays and potentially mitigating biofouling, a hydrogel CAB design was envisioned whose assembly is afforded by the electrostatic adhesivity of the wall (i.e., hollow rod) and caps (Figure 1). The CAB wall candidates were constructed as DN comb hydrogel, leveraging our prior work to adjust thermosensitivity and to reduce mesh size (Table 1) [14, 49, 56]. These were fabricated in a two‐step UV curing process, with the first network relatively more tightly crosslinked versus the second network based on BIS crosslinker levels. To create a more [−] anionic surface (for adhesivity to the cationic surfaces of the caps), the second network's ratio of NVP to [−] AMPS was systematically increased. To tune mesh size, [−] PAMPS_n_‐MA combs (*n* = 10 or 20) were incorporated into the first network. The DN hydrogel membrane compositions were grouped as “generations” and included two previously reported controls: *G_1_* (i.e., lacking in combs in first network; demonstrated rapid healing response and minimized fibrous capsule) [37] and *G_2_* (i.e., prepared with [−] PAMPS_10_‐MA combs at 3.75 mol% concentration in first network, and no AMPS in the second network; demonstrated retention of small molecules <5 nm) [49]. *G_3_‐series* and *G_4_*‐series were prepared with [−] PAMPS_10_‐MA (5.25 mol%) and [−] PAMPS_10_‐MA combs (2.625 mol%), respectively, at the noted mol% that represents the high concentration soluble in the precursor solution. While *G_3_* and *G_4_* were both prepared with no [−] AMPS in the second network, the *G_3_‐AMPS_x_* or *G_4_‐AMPS_x_* types were formed with varying ratios of NVP to [−] AMPS to create a more anionic hydrogel surface. Additionally, a PEG‐DA hydrogel (PEG) served as a non‐thermoresponsive, SN control.

### 2.2 Hydrogel CAB Wall – VPTT, Hydration, and Mechanical Properties

#### 2.2.1 VPTT

**FIGURE 2:**
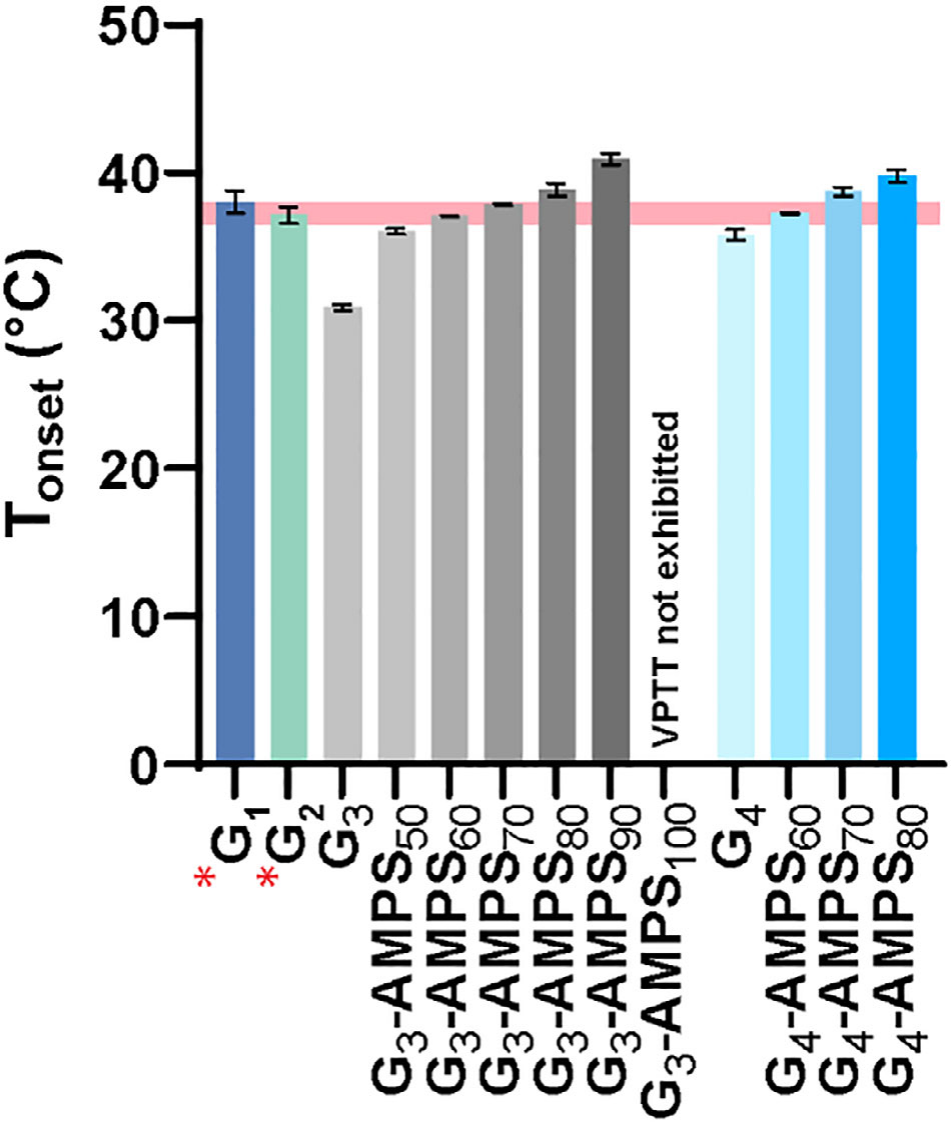
Onset VPPT (*T*_onset_) of DN hydrogels. * Denotes previously reported data (Dong et al., 2020). Pink region denotes the targeted *T*_onset_ (∼37°C–38°C).

Toward extending the longevity of sub‐Q glucose biosensors, we have established that ‘self‐cleaning’ thermoresponsive DN hydrogel membranes capable of cyclically deswelling and reswelling with local temperature fluctuations (above and below the VPTT, respectively) can mitigate cell attachment and accumulation [37, 38, 39, 57]. The precise VPTT *T_onset_* and *T_max_* are critical. The membrane should largely be in its swollen state (for enhanced biocompatibility and glucose diffusion), but slightly deswell with local temperature increases. Convenient for in vivo studies, temperature fluctuations exhibited in the sub‐Q rat tissue are commensurate to those displayed in the sub‐Q wrist tissue of a human. Thus, a *T_onset_* of ∼37°C–38°C (slightly higher than the sub‐Q wrist temperature, *T* ∼ 36°C) [58] is desirable [37, 38, 57]. Notably, *G_1_* (*T_onset_*, ∼37°C–38°C, and *T_max_*, ∼ 41°C) displayed minimal fibrous capsule formation (∼20–25 µm) after 90 days in a sub‐Q rat model [37]. As potential candidates for the CAB wall, this VPTT profile was therefore targeted for the *G_3_‐series* and *G_4_‐series* DN hydrogels. Versus *G_3_* and *G_4_* (i.e., no [−] AMPS in the second network), increased ratios of NVP to [−] AMPS for the *G_3_‐AMPS_x_* or *G_4_‐AMPS_x_* resulted in a relative increase in *T_onset_* values (Figure 2, Table S1). This was attributed to the greater hydrophilicity of [−] AMPS, which contains both amide and sulfo moieties, versus NVP, which contains only an amide moiety [59, 60]. *G_3_‐AMPS_60_, G_3_‐AMPS_70_, G_3_‐AMPS_80_*, and *G_4_‐AMPS_60_* all displayed a *T_onset_* within the target range (∼37°C–38°C). Given that the *T_onset_* of *G_4_‐AMPS_70_* exceeded the targeted range, compositions with even higher [−] AMPS in the second network were not explored (i.e., *G_4_‐AMPS_80,_ G_4_‐AMPS_90,_* and *G_4_‐AMPS*_100_).

#### 2.2.2 Equilibrium Water Content and Mechanical Properties

**FIGURE 3:**
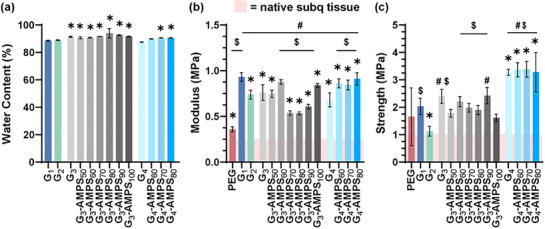
Physical properties of DN hydrogels: (a) water content, (b) compressive modulus (E_c_), and (c) compressive strength (*σ*_c_). *p < 0.05 versus G_1_. ^$^ *p* < 0.05 versus G_2_. ^#^ *p* < 0.05 versus PEG.

To potentially serve as the CAB wall, adequate hydration of the DN hydrogel is crucial for diffusivity, and also contributes to the reduction of the extent of the FBR [61]. All *G_3_‐series* and *G_4_‐series* DN hydrogels exhibited an EWC of ∼90%–94% (Figure 3a, Table S2). Versus *G_1_* (∼89%) and *G_2_* (∼91%), statistically higher EWC values (albeit not dramatically higher) were generally observed, particularly when [−] AMPS was introduced and increased in the second network, owing to the greater hydrophilicity of [−] AMPS versus NVP.

The CAB wall must be mechanically robust for sub‐Q injection (e.g., at the wrist) and indwelling. While hydrogels, particularly those with high water contents, are typically non‐rigid, weak, and brittle [62, 63, 64], DN hydrogels exhibited excellent compressive properties (Figure 3b,c, Table S3). Versus *PEG*, a greater compressive modulus (*E_c_*) is afforded by hydrophobic interactions (via NIPAAm), and electrostatic repulsive interactions (via [−] PAMPS_n_‐MA and [−] AMPS) that induce chain stiffening effects [65, 66]. Enhanced compressive strength (*σ_c_*) as well as toughness (*U_c_*) was achieved by stress dissipation effects imparted by the DN hydrogels’ asymmetrically crosslinked networks [67], and the sacrificial network formed by hydrogen bonds [68] between [−] PAMPS_n_‐MA combs and NIPAAm, and the ‘dynamic crosslinks’ (e.g., hydrophobic and electrostatic interactions, and hydrogen bonds). In our prior study, *G_1_* cylindrical rods (i.e., no [−] PAMPS_n_‐MA and no [−] AMPS) were successfully implanted via trocar needle into the sub‐Q tissue of rats [37, 69]. Its strength (*σ_c_* ∼ 2 MPa) contributed to this and was expected to tolerate forces experienced in the upper extremities (∼1 MPa) [69]. While its *E_c_* (∼0.9 MPa) somewhat exceeded that of the surrounding sub‐Q and dermis tissues (*E_c_ *∼ 0.02–0.5 MPa), a scenario that can magnify the FBR [70, 71], this was not observed and was attributed to its self‐cleaning nature. For *G_2_* (i.e., [−] PAMPS_10_‐MA at 3.65 mol%, no [−] AMPS in the second network), there was a reduction in *E_c_* (∼0.7 MPa) and *σ_c_* (∼1.1 MPa) versus *G_1_*. For the *G_3_‐series* (i.e., [−] PAMPS_10_‐MA at 5.25 mol%), the concentration of [−] AMPS in the second network impacted *E_c_* values (∼0.5–0.88 MPa). Relatively higher *E_c_* values (∼0.7–0.91 MPa) were observed for *G_4_‐series* (i.e., [−] PAMPS_20_‐MA at 2.625 mol%). This indicates that the longer comb macromers may be more effective in producing a chain stiffening effect arising from electrostatic repulsive interactions. Despite displaying slightly larger *E_c_* values (∼0.50–1 MPa) than sub‐Q and dermis tissue, all compositions displayed significantly lower *E_c_* than those of on‐the‐market CGMs (*E_c_*: 3–200 GPa) [72]. Resultingly, the developed DN hydrogels are expected to exhibit minimal adverse shear stresses responsible for FBR. As previously noted, the compressive strength of *G_2_* (*σ_c _* ∼ 1 MPa) was lower compared to *G_1_* (*σ_c _* ∼ 2 MPa), attributed to the former's increased repulsive electrostatic interactions, which disrupt stress dissipation afforded by the asymmetric DN design [49]. Compared to *G_1_*, greater *σ_c_* values were observed for the *G_3_‐series* (∼1.9–2.4 MPa) and particularly for the *G_4_‐series* (∼3.3–3.4 MPa). As with stiffness, the longer combs were most effective in increasing strength, perhaps due to superior stress dissipation by longer chains. Overall, all *G_2_‐series* and *G_3_‐series* hydrogels display robust mechanical properties, including toughness (*U_c_*) and significant strain at failure (*ε_c_*) of ∼42%–55%. The DN hydrogels also exhibited robust mechanical properties when tested in tension (Figure S2, Table S4). For the *G_3_‐series* and *G_4_‐series* hydrogels, the incorporation of [−] AMPS in the second network likewise resulted in robust tensile modulus (*E_t_*) and tensile strength (*σ_t_*) values. The strength of *G_4_‐series* (*σ_t_* ∼ 195–600 kPa) hydrogels was higher than that of *G_3_‐series* hydrogels (*σ_t_* ∼ 85–150 kPa). Percentage strain at failure (*ε_t_*) and tensile toughness (*U_t_*) values were also notably higher for *G_4_‐series* (∼21%–42%; *U_t_ *∼ 20–88 kJ m^−3^) versus *G_3_‐series* hydrogels (∼15%–21%; *U_t_ *∼ 7–14 kJ m^−3^).

### 2.3 Hydrogel CAB Wall – Diffusivity

#### 2.3.1 Mesh Size

The DN hydrogel that comprises the CAB wall must retain (i.e., prevent the diffusion) of the small‐sized optical glucose assay molecules contained within its hollow cavity while allowing diffusion of glucose (*D_h_* ∼ 0.8 nm) from the surrounding ISF. The mesh size (ξ) of DN hydrogels could not be determined via conventional methods (e.g., scanning electron microscopy and atomic force microscopy) [73, 74] due to its asymmetric DN and presence of combs. Thus, FITC‐dextran permeability studies were conducted, and utilized FITC‐dextrans (molecular weight ∼4k, 10k, 20k, and 40k g/mol) that corresponded to different low hydrodynamic diameters (*D_h_* ∼3, 4, 7, and 10 nm, respectively). A hydrogel disc was sequentially soaked in a designated FITC‐dextran solution (0.01 mg/mL), moved to fresh DI, and the FITC‐dextran released via diffusion quantified. In this way, a FITC‐dextran with a *D_h_* < hydrogel mesh size would diffuse into and out of the hydrogel. An aliquot of the resulting solution was removed, and its fluorescence intensity was determined. The resulting solution's fluorescence intensity value was then correlated to the concentration of FITC‐dextran that diffused through the hydrogel using a calibration curve (Figure S3). Diffusion was considered negligible if the calculated concentration was ≤0.00004 mg/mL (i.e., that of DI water).

**FIGURE 4:**
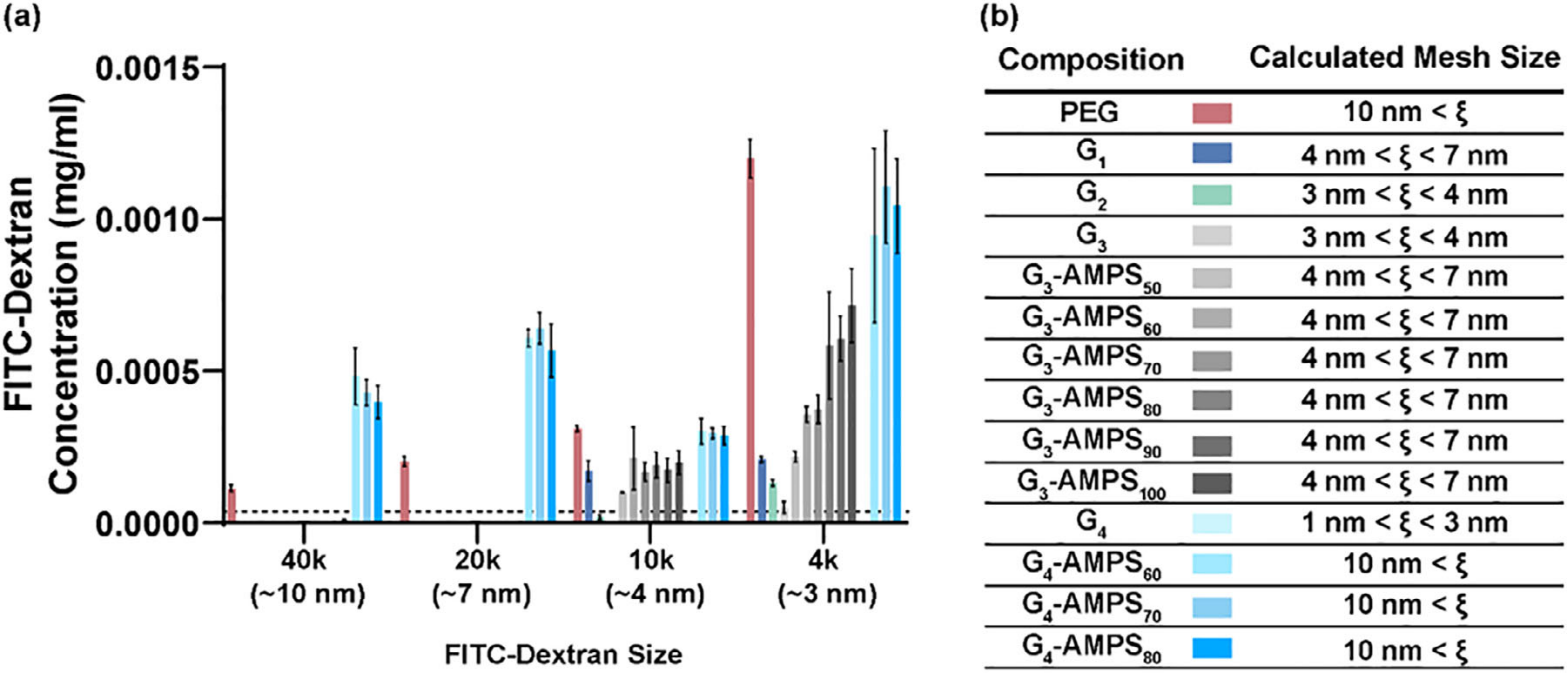
DN hydrogels: (a) Size exclusion study of hydrogel discs (22°C). The dashed line represents a negligible concentration of FITC‐dextran (≤0.00004 mg/mL). (b) Calculated mesh sizes (*ξ*) (22°C).

The first to be determined were the mesh sizes of the controls: *PEG*, *G_1_* (first network: no combs, second network: no [−] AMPS), and *G_2_* (first network: [−] PAMPS_10_‐MA at 3.25 mol%, second network: no [−] AMPS) (Figure 4). Versus *PEG* (*ξ* > 10 nm), the mesh size of *G_1_* was lower (4 nm < *ξ* < 7 nm), and was further reduced for *G_2_* (3 nm < *ξ* < 4 nm) owing to the presence of combs that sterically block the mesh “windows” [49]. However, as described later, *G_1_* and *G_2_* lack adhesivity to the *semi‐IPN‐PA/[+]PE* (i.e., CAB cap) such that, as CAB walls, their cavity ends would not be adequately sealed. Thus, the mesh sizes were determined for the *G_3_‐series* (first network: [−] PAMPS_10_‐MA at 5.25 mol%, second network: variable [−] AMPS). The mesh size of *G_3_* was not changed versus that of *G_2_* (both without [−] AMPS in the second network), indicating that the relative increase in concentration of [−] PAMPS_10_‐MA of *G_3_* did not cause greater blockage of mesh windows. When [−] AMPS was introduced to the second network (i.e., *G_3_‐AMPS_x_*), irrespective of concentration, the mesh size was increased slightly (4 nm <* ξ* < 7 nm). It is hypothesized that the inclusion of [−] AMPS led to greater electrostatic repulsion, causing expansion of the mesh windows such that the [−] PAMPS_10_‐MA combs were slightly less effective. Next, the mesh sizes were determined for the *G_4_‐series* (first network: PAMPS_20_‐MA, second network: no AMPS). For *G_4_* (i.e., no [−] AMPS in the second network), the mesh size was very low (1 nm < *ξ* < 3 nm). The inclusion of [−] AMPS to the second network (i.e., *G_4_‐AMPS_x_*) led to an appreciably greater mesh size (10 nm < *ξ*). The higher mesh sizes of *G_4_‐AMPS_x_* versus *G_3_‐AMPS_x_* hydrogels may be due to the lower concentration of the longer combs (2.625 mol%) versus shorter combs (5.25 mol%), respectively.

#### 2.3.2 Glucose Diffusion

**FIGURE 5:**
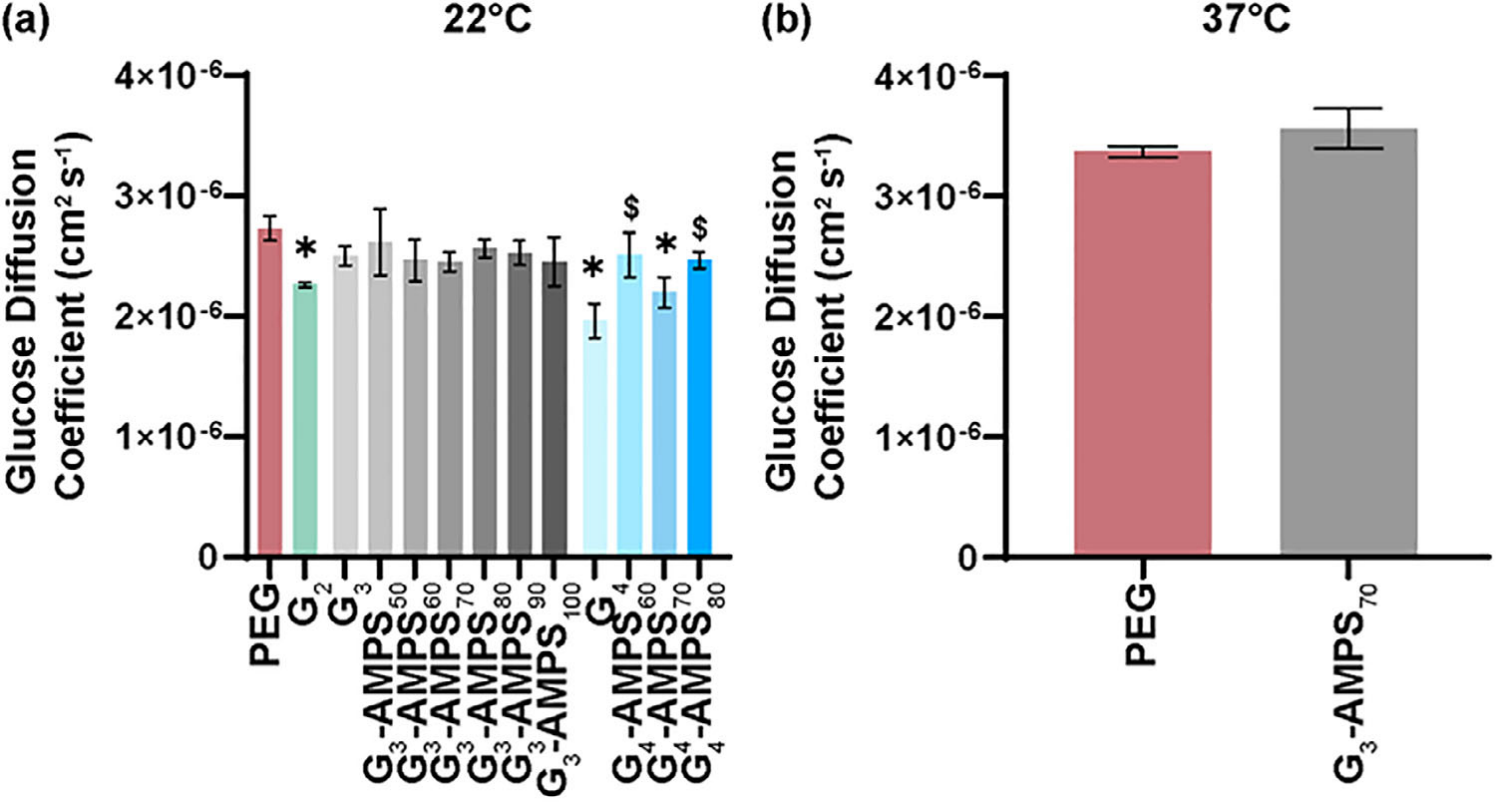
Glucose diffusion coefficient (*D*) at (a) 22°C, and (b) 37°C. *p < 0.05 vs *PEG*. ^$^ *p* < 0.05 vs corresponding composition without [−] AMPS in second network (i.e., *G_3_* or *G_4_*).

The diffusion of glucose (*D_h_* ∼ 0.08 nm) of the ISF must readily occur through the DN hydrogel that comprises the CAB wall. An acceptable hydrogel glucose diffusion coefficient (*D*) value was considered to be that (or greater) of glucose diffusion through sub‐Q tissue or a fibrous capsule (*D* ∼ 2  ×  10^−6^ cm^2^ s^−1^) [75, 76]. Glucose diffusion experiments were first conducted at 22°C, representing a temperature that DN hydrogels are in a fully swollen state (Figure 5a, Table S5). Both the non‐thermoresponsive control, *PEG* (*D* $∼\;2.75\;\times \;10^{-6}\;\;cm^{2}\;s^{-1}$) and all DN hydrogels (*D* ∼ 2  ×  10^−6^ cm^2^ s^−1^ to 3  ×  10^−6^ cm^2^ s^−1^) were within the desirable range. At 37°C, *D* of PEG increased ($∼3.37\;\times \;10^{-6}\;cm^{2}\;s^{-1}$), likely due to greater Brownian motion (Figure 5b, Table S6). In the case of DN hydrogels, 37°C represents the onset of deswelling. Given its desirable mesh size (and adhesivity to the CAB cap as discussed later), glucose diffusion of *G_3_‐AMPS_70_* at 37°C was evaluated, and *D* determined to be ∼3.5  ×  10^−6^ cm^2^ s^−1^. Thus, in the slightly deswollen state, glucose diffusivity was retained.

### 2.4 Hydrogel CAB Cap – Fabrication and Properties

To create a hydrogel CAB cap to seal the ends of the CAB wall, electrostatic attractive forces were leveraged. As described above, to potentially serve as CAB walls, DN hydrogels with a negatively charged surface were created by the introduction of [−] AMPS into the second network. Thus, a CAB cap was created with a hydrogel (*semi‐IPN‐PA/[+]PE*) having a cationic surface, afforded by a design semi‐IPN comprised of a polyampholyte crosslinked network and cationic [+] polyelectrolyte (PADAMC). The zwitterionic nature of polyampholyte hydrogels endows them with adaptive adhesion through electrostatic interactions and the ability to adhere in wet environments, making them attractive candidates for biomedical use [53, 54, 77, 78, 79]. The charge‐balanced polyampholyte crosslinked network was developed based on prior reports [77, 80]. It further included a cationic polyelectrolyte ([+] PDADMAC) whose uncrosslinked nature was expected to improve electrostatic adhesivity by increasing chain mobility. This *semi‐IPN‐PA/[+]PE* was successfully prepared via a 1‐step UV cure of BIS crosslinker, [+] NaSS (1.2 m), [−] AETAC (1.3 m), [+] PDADMAC (*M_w_* < 100k; 3 wt.% w.r.t total monomer). The *semi‐IPN‐PA/[+]PE* hydrogel CAB caps displayed hydration (EWC ∼ 50%) similar to typical values for traditional PA hydrogels (EWC ∼ 40%–60%) (Table S2) [63]. The electrostatic, semi‐IPN design also imparted robust mechanical properties relative to the *PEG* control (Table S3, Table S4).

### 2.5 Hydrogel Adhesivity

**FIGURE 6:**
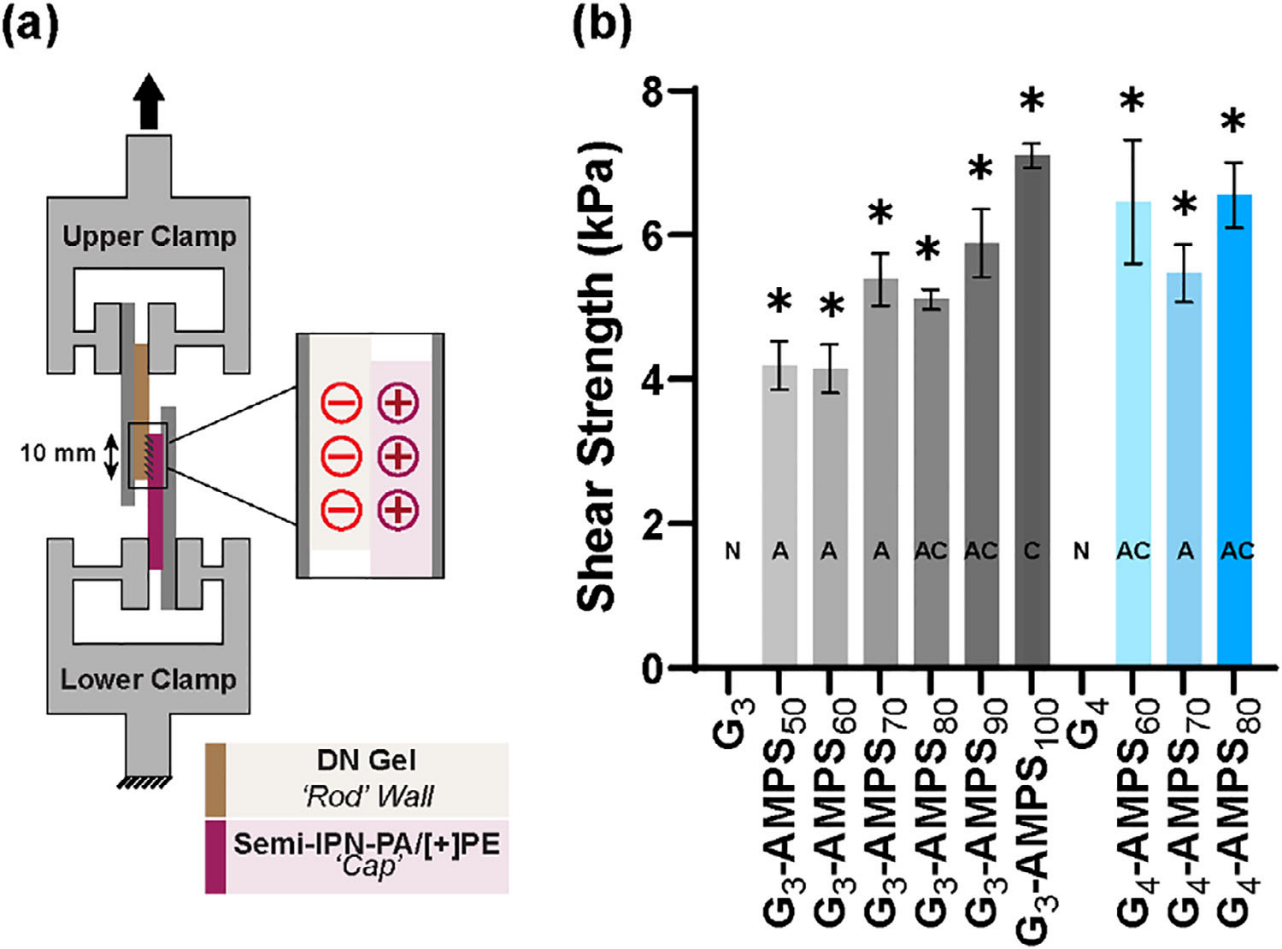
Lap shear test (a) test configuration, and (b) shear strength between DN hydrogels (i.e., CAB wall) and the sIPN‐PA (i.e., CAB cap). Mode of failure between contact gels indicated as: ‘N’ [no adhesion]; ‘A’ [adhesive failure]; ‘AC’ [both adhesive and cohesive failure]; and ‘C’ [cohesive failure]. *p < 0.05 vs corresponding composition with no AMPS in the second network (i.e., G_3_ or G_4_).

The ability of the DN hydrogels (i.e., CAB wall candidates) to adhere to the *semi‐IPN‐PA/[+]PE* hydrogel (i.e., CAB cap) via electrostatic attractive forces was assessed via interfacial shear strength measurements. The designated DN hydrogel and the *semi‐IPN‐PA/[+]PE* hydrogel were formed as planar rectangular specimens and contacted in a 10 mm × 10 mm overlap (Figure 6a). The construct was displaced at a rate of 100 mm min^−1^ due to the known highly extensible nature of polyampholyte hydrogels [77]. In addition to the measured shear strength (kPa), the nature of the failure was noted (i.e., none, adhesive, cohesive, or adhesive and cohesive) (Figure 6b, Table S7). Because *semi‐IPN‐PA/[+]PE* was highly extensible in tension (*ε_t _∼ *551%, Table S4), as adhesivity increased, the construct was able to withstand the associated greater forces without failing prematurely (Table S7, Figure S4). Despite [−] PAMPS_n_‐MA in the first networks but having no [−] AMPS in the second network, *G_3_* and *G_4_* did not adhere toward the cationic *semi‐IPN‐PA/[+]PE* hydrogel.

In contrast, the incorporation of AMPS into the second network successfully resulted in adhesion of *G_3_‐AMPS_x_* and *G_4_‐AMPS_x_* hydrogels to the *semi‐IPN‐PA/[+]PE* hydrogel. This was expected, as the outermost (‘last’) network is known to dictate the electrostatic surface properties of multi‐network hydrogels [51]. The adhesive shear strength of the *G_3_* and *G_4_* type hydrogels followed trends similar to those in tension. For *G_3_* series hydrogels, as [−] AMPS increased in the second network (from 50% to 100%), the interfacial shear strength with the cationic *semi‐IPN‐PA/[+]PE* hydrogel increased owing to a more negatively charged surface. Shear strength was the highest for *G_3_‐AMPS_100_* (∼7 kPa), and was the only DN hydrogel to exhibit cohesive failure. However, incorporating AMPS into the second network of the DN design ensured all DN hydrogels would readily adhere toward the *semi‐IPN‐PA/[+]PE ‘caps’*, enabling the construction of a CAB. The adhesive shear strength of the connection between hydrogels is promising within DI water at RT. Electrostatic interactions at physiologic conditions may impact the adhesive shear strength of the material due to charge screening afforded by salts [81], and future work will explore this potential impact.

### 2.6 CAB Construction and Diffusion

**FIGURE 7:**
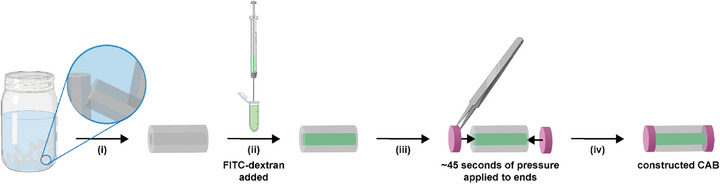
CAB assembly via sequential steps: (i) Hollow DN hydrogel rods (∼2.5 mm × ∼5 mm, OD × l) were removed from DI water and blotted with a Kim wipe, which also wicked water from the central cavity; (ii) FITC‐dextran solution (i.e., liquid glucose sensing assay mimic) was loaded into the cavity; (iii) semi‐IPN‐PA/[+]PE hydrogel ‘caps’ (∼2.5 mm × ∼1 mm, *d* × *t*) were manually held on the ends of the for ∼45 s, and (vii) resulted in the final CAB.

**FIGURE 8:**
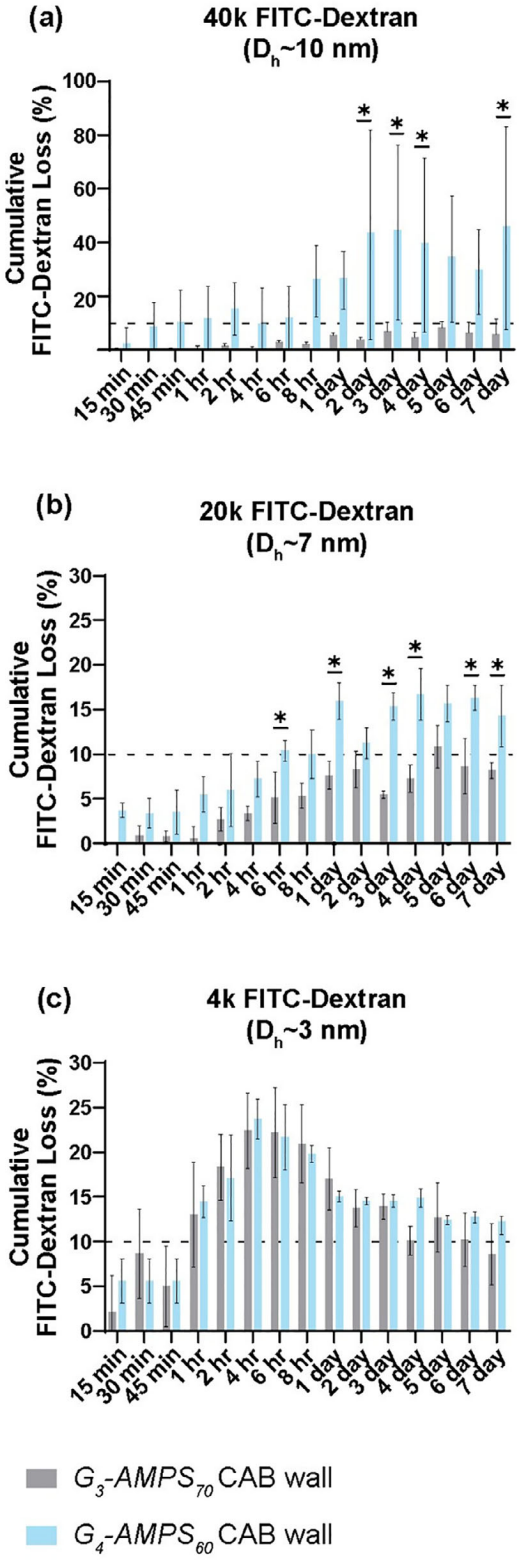
CABs were constructed using *G_3_‐AMPS_70_* (4 nm < *ξ* < 7 nm) [grey] or *G_4_‐AMPS_60_* (10 < *ξ*). [blue] as CAB walls (i.e., hollow rods containing designated FTIC‐dextran solution) and sealed with *semi‐IPN‐PA/[+]PE* CAB caps. Cumulative FITC‐dextran loss out of the CABs was determined for FITC‐dextran of various sizes: (a) 40k g/mol (*D_h_* ∼ 10 nm), (b) 20k g/mol (*D_h _* ∼ 7 nm), and (c) 4k g/mol (*D_h_* ∼ 3 nm). The dashed line represents a 10% loss of FITC‐dextran. *p < 0.05 versus the corresponding composition at the same time point.

The *G_3_‐AMPS_70_* DN hydrogel was identified as the optimal candidate to serve as CAB wall based on: (i) a VPTT profile in the targeted range, (ii) robust mechanical properties, (iii) a mesh size that could retain small molecules (4 nm < *ξ* < 7 nm), (iv) glucose diffusivity, and (v) excellent adhesivity to the *semi‐IPN‐PA/[+]PE* hydrogel (i.e., CAB cap). Thus, *G_3_‐AMPS_70_* DN hydrogels were formed as hollow rods, their cavities filled with solutions of FITC‐dextran, and their ends sealed with *semi‐IPN‐PA/[+]PE* hydrogel caps (Figure 7). As a control group with less potential to limit small molecule diffusion, CABs were likewise assembled with *G_4_‐AMPS_60_* walls owing to their higher mesh size (10 <* ξ*). Representing small molecule assays of varying sizes, three FITC‐dextran solutions (10 wt.%) [∼4k g/mol; *D_h_ *∼ 3 nm; 20k g/mol; *D_h_ *∼ 7 nm; and 40k g/mol; *D_h_* *∼ *10 nm] were placed into the central cavity of the CAB wall, and the quantity that leached out over a 7‐day period determined. Owing to a higher mesh size, greater loss of FITC‐dextrans was observed for CABs comprised of the *G_4_‐AMPS_60_* (10 < *ξ*) versus *G_3_‐AMPS_70_* (4 nm < *ξ* < 7 nm) CAB walls (Figure 8). For those based on *G_4_‐AMPS_60_*, in just hours, >10% of 40k g/mol (*D_h _∼ *10 nm) as well as 20k g/mol (*D_h _∼ *7 nm) FITC‐dextrans were lost. By 2 days, this reached ∼50% cumulative loss for the 40k FITC‐dextran. In contrast, after 7 days, CABs based on *G_3_‐AMPS_70_* lost <10% of 40k and 20k g/mol FITC‐dextrans. As predicted based on their mesh sizes, >10% of 4k FITC‐dextran (*D_h _∼ *3 nm) was lost for CABs comprised of either *G_3_‐AMPS_70_* or *G_4_‐AMPS_60_*. Owing to its small size (*D_h_ *∼ 0.8 nm), it is expected that glucose will readily diffuse through the network, and was subsequently confirmed via determination of glucose diffusion coefficients. Overall, *G_3_‐AMPS_70_* demonstrated its effectiveness to serve as the CAB wall based on its ability to retain small molecules (∼7–10 nm) within its hollow cavity, both by limiting diffusion as well as by effectively being sealed by the *semi‐IPN‐PA/[+]PE* CAB cap.

## 3 Conclusion

Membrane diffusivity is an integral aspect to consider in the development of a hydrogel to prepare a sub‐Q glucose biosensor that houses optical glucose‐sensing assays. Our previous work established the development of “self‐cleaning” comb DN hydrogels that are able to limit biofouling via cyclical deswelling/reswelling in response to local temperature fluctuations, and to retain small molecules (*D_h _* < 10 nm). Herein, we developed “CABs” or cargo‐carrying adhesive biosensors comprised of an adhesive “self‐cleaning” DN hollow hydrogel rod (whose central cavity can retain an optical assay) and hydrogel caps that seal the ends of the rod. The CAB wall and caps were assembled via electrostatic attractive interactions stemming from their respective anionic and cationic surfaces. CAB wall candidates were developed by incorporating [−] PAMPS_n_‐MA (*n* = 10 for *G_3_‐series* and *n* = 20 for *G_4_‐series*) comb macromer within the first network to tune the mesh size of the hydrogel, and introducing [−] AMPS into the second network at varying concentrations to create a more anionic surface. The CAB cap was formed from a semi‐IPN of crosslinked polyampholyte and non‐crosslinked cationic polyelectrolyte (*semi‐IPN‐PA/[+]PE*). For the CAB wall candidates, while greater [−] AMPS content resulted in a relative increase in the *T_onset_*, several compositions displayed the necessary *T_onset_* (∼37°C–38°C) to ensure self‐cleaning could occur within sub‐Q tissue. Overall, the DN design and incorporation of [−] PAMPS_n_‐MA and [−] AMPS imbued the hydrogels with robust mechanical properties despite high hydration (∼90%), including particularly high compressive strength values (∼1.6–3.3 MPa). Additionally, all CAB wall compositions displayed good mechanical rigidity (*E_c_* ∼ 0.5–1 MPa), but values were substantially lower than those of on‐the‐market transcutaneous CGM electrodes (typically in the GPa range) that could lead to an enhanced FBR. The simultaneous glucose diffusion and retention of small assay molecules by the CAB wall is critical. All CAB wall candidates achieved the target glucose diffusivity (*D* ∼ 2  ×  10^−6^ cm^2^ s^−1^); however, their mesh sizes varied. *G_3_‐AMPS_x_* hydrogels (first network: [−] PAMPS_10_‐MA; second network: [−] AMPS) displayed lower mesh size values (4 nm <* ξ* < 7 nm) versus *G_4_‐AMPS_x_* hydrogels (first network: [−] PAMPS_20_‐MA; second network: [−] AMPS) (10 nm < *ξ*). Per lap shear tests, all DN hydrogels containing [−] AMPS in the second network were adhesive to the *semi‐IPN‐PA/[+]PE* hydrogel. A CAB was constructed with a CAB wall based on a DN hydrogel (*G_3_‐AMPS_70_*) that displayed the targeted properties, and FITC‐dextran solutions housed in the central cavity were used to showcase the successful retention (∼90%) of small molecules (*D_h_ ∼ *7 nm and *D_h_* *∼ *10 nm) over 7 days. Overall, this CAB design represents a promising approach to house emerging optical assays, enabling the development of sub‐Q glucose biosensors.

## 4 Experimental Section

### 4.1 Materials

*N*‐Isopropylacrylamide (NIPAAm, 97%), 2‐acrylamido‐2‐methylpropane sulfonic acid ([−] AMPS, 97%), *N*‐vinylpyrrolidone (NVP, ≥99%), *N, N’*‐methylenebisacrylamide (BIS, 99%), 2‐hydroxy‐4’‐(2‐hydroxyethoxy)‐2‐methylpropiophenome (Irgacure 2959, 98%), methacrylic anhydride (MA, 94%), sodium hydroxide (NaOH, ≥97%), hydrochloric acid (HCl, 37%), cysteamine hydrochloride (AET, ≥98%), ammonium persulfate (APS, ≥ 98%), trimethylamine (Et_3_N), poly(ethylene glycol) (PEG, molecular weight = 3350 g/mol), potassium carbonate (K_2_CO_3_), magnesium sulfate (MgSO_4_), sodium bicarbonate (NaHCO_3_), potassium phosphate dibasic (K_2_HPO_4_, anhydrous ≥99%), potassium dihydrogen phosphate (KH_2_PO_4_, ≥98%), fluorescein isothiocyanate‐dextran (FITC‐dextran, 4k, 10k, 20k, and 40k g/mol), D‐(+)‐glucose (≥99.5%), sodium 4‐vinylbenzenesulfonate (NaSS, ≥90%), [2‐(acryloyloxy)ethyl] trimethyl‐ammonium chloride solution (AETAC, 80 wt.% in H_2_O), poly(diallyldimethylammonium chloride) solution ([+] PDADMAC, average *M_w_* < 100k, 35 wt.% in H_2_O), 2‐oxoglutaric acid (2‐oxo), deuterium oxide (D_2_O, NMR grade), ethanol (HPLC grade), and acetone (ACS Reagent, ≥99%) were obtained from Millipore‐Sigma. Phosphate‐buffered saline (PBS, 1×, pH 7.4, without calcium and magnesium, Corning) was obtained from Fisher Scientific. All chemicals were used directly without further purification. Deionized (DI) water with a resistance of 18.2 MΩ·cm was purified with a Barnstead E‐Pure ultrapure water purification system, Thermo Scientific.

### 4.2 Comb Macromer Synthesis

All reactions were conducted in an inert nitrogen (N_2_) environment with a Teflon‐coated stir bar. Foil was wrapped around a bottom flask (rbf) to protect reactions from light as noted. Product structures were verified through end‐group analysis via ^1^H‐NMR spectroscopy using a Bruker Avance Neo 400 MHz spectrometer with an Ascend magnet and an automated tuning 5 mm broadband probe, operating in the Fourier transform mode with D_2_O as the standard.

### 4.3 PAMPS_n_‐MA Comb Macromer (*n* = 10, *n* = 20)

The [−] PAMPS_n_‐MA comb macromers were synthesized as previously reported in the literature with slight modifications [49]. First, PAMPS_n_‐NH_2_ was synthesized as follows. [−] AMPS (5.18 g, 25 mmol) was added to a 1 m NaOH solution (25 mL). The pH of the solution was adjusted to between 4 and 5 with KH_2_PO_4_ (3.4 g, 25 mmol) and K_2_HPO_4_ (0.84 g, 4.82 mmol) and was then sparged with N_2_ for 15 min at room temperature (RT). The reaction was initiated using a redox couple system consisting of APS (initiator) (0.57 g, 2.50 mmol) and AET (chain transfer agent) (0.57 g, 5.02 mmol). APS and AET were added to the solution at a 1:2 molar ratio. The ratio of [−] AMPS to AET (*n* = 10, 10:1, *n* = 20, 20:1) was used to control the degree of polymerization (*n*). The solution was sparged with N_2_ for an additional 10 min, and subsequently heated to 60°C. The solution was then sparged for 10 min and purged for 5 min. The reaction proceeded overnight (ON) under positive N_2_ pressure, and was terminated by adjusting the pH to ∼8 with 1 m NaOH. [−] PAMPS_n_‐NH_2_ was recovered and purified via precipitation into ethanol. The precipitated [−] PAMPS_n_‐NH_2_ was dried in vacuo (RT, 30 in. Hg) for 2 days. Next, [−] PAMPS_n_‐NH_2_ was methacrylated using methacrylic anhydride (MA). Purified [−] PAMPS_n_‐NH_2_ ([1.5 g (*n = *10), 3 g (*n *= 20)], 0.75 mmol) was dissolved in 7.5 mL of PBS, and the pH of the solution was adjusted to ∼7 with 1 m HCl. The solution was sparged and purged with N_2_ for 30 and 10 min, respectively. MA (112.5 µL, 0.75 mmol) was slowly added dropwise to the solution and was subsequently heated to 60°C. The reaction proceeded ON under positive N_2_ pressure. [−] PAMPS_n_‐MA was purified via precipitation into ethanol, and dried in vacuo (RT, 30 in. Hg) for 2 days. It was then reprecipitated into acetone and the final product dried *in vacuo* (RT, 30 in. Hg) for 2 days.

### 4.4 Hydrogel ‘CAB Wall’ Fabrication

Representing the wall of the CAB, double network (DN) hydrogels were prepared as both planar sheets (for material characterization) and hollow rods (for CAB construction). A 2‐step UV curing process was employed, wherein a single network (SN) hydrogel was prepared and subsequently soaked in a DN precursor solution, and then UV‐cured to form either a DN hydrogel sheet or rod. The compositions, grouped as “generations” (i.e., *G_1_*, *G_2_*, *G_3_*, and *G_4_*), are described in Table 1, with the previously reported *G_1_* [37] and *G_2_* [49]serving as controls. The aqueous SN precursor solution was prepared similarly to previous reports [37, 49]. For *G_3_‐series* and *G_4_‐series* hydrogels, these were prepared as follows. The SN precursor solution consisted of a 1.11 m total monomer concentration of NIPAAm, [−] AMPS, and [−] PAMPS_n_‐MA. The NIPAAm concentration was maintained at 85 mol%, and the remaining 15 mol% was comprised of [−] AMPs and [−] PAMPS_n_‐MA, specifically [−] PAMPS_10_‐MA for *G_3_‐series* and [−] PAMPS_20_‐MA and *G_4_‐series*. The concentration of [−] PAMPS_n_‐MA introduced into the first network was the highest mol% that was soluble in the precursor solution: 5.25 mol% [−] PAMPS_10_‐MA (*G_3_‐series*) and 2.625 mol% [−] PAMPS_20_‐MA (*G_4_‐series*). The rbf containing the SN precursor solution was wrapped in foil, and BIS crosslinker (3.3 mol% w.r.t. total monomer concentration), and Irgacure 2959 photoinitiator (4.6 mol% w.r.t. total monomer concentration) subsequently added. The second network precursor solution was comprised of NIPAAm (2.5 m), [‐] AMPS, NVP, BIS crosslinker (0.15 mol% w.r.t. NIPAAm concentration), and Irgacure 2959 photoinitiator (2 mol% w.r.t. NIPAAm concentration). While maintained at a combined total of 5% w/v, the ratio of NVP to [−] AMPS was varied: *G_3_‐series* (50:50, 40:60, 30:70, 20:80, 10:90, and 0:100 wt.%), and *G_4_‐series* (40:60, 30:70, and 20:80 wt.%). The rbf was wrapped with foil prior to the addition of BIS and Irgacure. *G_3_‐series* and *G_4_‐series* compositions are denoted as “*G_3_‐AMPS_x_*” or “*G_4_‐AMPS_x_*” where *x* is the % of [−] AMPS in the second network precursor solution (Table 1). Fabrication for both the DN planar sheets and hollow rods is described below.

### 4.5 DN Hydrogel Planar Sheet Fabrication (CAB Wall)

DN hydrogel sheets (∼2 mm post‐swelling thickness) were prepared as follows. The SN precursor solution was injected into a rectangular sandwich mold consisting of a 1 mm silicone spacer clamped between 2 rectangular glass slides (75 mm × 50 mm). The mold was placed into an ice–water bath to ensure NIPAAm monomer remained soluble during exposure to UV light (UV‐transilluminator, 6 mW/cm^2^, *λ*_peak_ = 365 nm). The mold was exposed for 30 min, rotating the mold after 15 min. The cured SN hydrogel was removed from the mold and soaked in the DN precursor solution at RT for 2 days. The SN hydrogel was then placed into a second rectangular sandwich mold separated by polycarbonate spacers (*t *∼ 1.25 mm) and UV‐cured for 30 min as described above. The fabricated DN hydrogel was then placed in DI water, with daily exchanges occurring for 3 sequential days following the initial soak. Planar sheets (*t* ∼ 1.8 mm) were used to determine the thermosensitivity, equilibrium water content, mechanical properties, mesh size, and glucose diffusion coefficient.

### 4.6 DN Hydrogel Hollow Rod Fabrication (CAB Wall)

DN hydrogel hollow rods (∼2.5 mm × ∼5 mm post‐swelling, *OD* × *l*) were prepared by injecting the SN precursor solution into a cylindrical glass mold (6 mm × ∼1 mm × ∼20 mm, *OD* × *ID* × *l*). The ends of the cylindrical mold were sealed with Parafilm, and Teflon‐coated caps with a hole (*d* ∼ 400 µm) were placed at the ends. A stainless‐steel wire (∼400 µm, outer diameter) was inserted through the Teflon‐coated caps to form a hollow central cavity (Figure S1). The assembled cylindrical mold was placed into an ice–water bath, and exposed to UV light for 30 min [37]. The resulting SN hollow hydrogel rods were slightly dehydrated to remove them from the cylindrical mold. The assembled cylindrical mold and hydrogel were placed into an oven (120°C, 10 min). Once removed from the molds, the hollow SN hydrogel rods were soaked in a DN precursor solution (RT, 2 days). Next, a 500‐µm stainless steel wire was inserted into the central cavity and tightly wrapped with cling film, ensuring a flush surface, and subsequently exposed to UV light for 10 min, rotating 180° after 5 min. Following the removal of the cling film, the resulting hollow DN hydrogel rod was placed into DI water with daily exchanges occurring for 3 days. Hollow rods served as CAB walls to assemble CABs (i.e., with CAB caps) and subsequently used for FITC‐dextran leaching studies. The final hydrogel rods were cut to size using a razor blade with final dimensions of (∼2.5 mm × ∼1 mm × ∼5 mm, *OD × ID × l*).

### 4.7 Poly(ethylene glycol)diacrylate (PEG‐DA) Hydrogel Fabrication

For comparisons to DN hydrogels, a PEG‐DA hydrogel control (*PEG*) was fabricated as previously reported with slight modifications [49]. A photoinitiator solution was prepared by dissolving Irgacure 2959 photoinitiator (30% w/v) in NVP, and 30 µL of the photoinitiator solution was subsequently added into a precursor solution consisting of 10% w/v PEG‐DA (0.3 g) in DI (3 mL). The resulting solution was injected into a sandwich mold with a 1.75 mm silicone spacer, and was exposed to UV light for 14 min, rotating the mold at 7 min. The PEG‐DA hydrogel was placed in DI with daily water exchanges occurring for 3 days at RT prior to any testing.

### 4.8 Hydrogel ‘CAB Cap’ Fabrication

A semi‐IPN‐PA/[+]PE hydrogel was fabricated as follows, with the crosslinked polyampholyte network portion prepared analogous to that reported by Gong et al. [54, 80]. DI, [−] NaSS (52 mol%), [+] AETAC (48 mol%), and the [+] PDADMAC (3 wt.% w.r.t respect to total monomer concentration) were added to a rbf (wrapped in foil), and stirred until dissolved. NaSS and AETAC were incorporated at a total monomer concentration of 2.5 m. BIS crosslinker (0.3 mol% w.r.t total monomer concentration) and 2‐oxo photoinitiator (0.1 mol% w.r.t total monomer concentration) were then added to the rbf and stirred until dissolved. The solution was sparged with N_2_ for 10 min, and subsequently placed under vacuum for 10 min with constant stirring via stir bar to remove air from the solution. The resulting solution was injected into a rectangular mold comprised of a 1 mm thick silicone spacer between 2 glass slides and held together with clips. Curing was conducted by exposing the molds to UV light (UV‐transilluminator, 6 mW/cm^2^, *λ*_peak_ = 365 nm) ON (∼16 h). The resulting hydrogel was removed from the mold and submerged in DI at RT with daily water exchanges for one week to remove any unincorporated starting material. Planar sheets (*t* ∼ 1 mm) were used to determine the equilibrium water content, and mechanical properties. CAB caps (2.5 mm × ∼1 mm, *d* × *t*) were collected with a biopsy punch.

### 4.9 Equilibrium Water Content (EWC)

Hydrogel discs (6 mm × ∼1.8 mm, *d* × *t*; *N *≥ 3) were collected from different water‐swollen hydrogel sheets using a biopsy punch. A Kim Wipe was used to blot the surface of the disc, weighed to determine its swollen weight (*W_s_*), and subsequently dried *in vacuo* overnight (RT, 30 in. Hg) to obtain the dry weight (*W_d_*). Equilibrium water content (*W_c_*) was determined from Equation (1).

(1) $$\begin{array}{c} \;W_{c}=\frac{W_{s\;}\;-W_{d}}{W_{s}}\times 100 \end{array}$$

### 4.10 Volume Phase Transition Temperature (VPTT)

Hydrogel VPTT profiles were determined using differential scanning calorimetry (DSC, TA Instruments Q100). A swollen hydrogel sample (∼10 mg, *N* ≥ 3) was blotted with a Kim Wipe and subsequently sealed in an aluminum hermetic pan. The temperature was cooled to 0°C, increased to 60°C, and returned to 0°C for 2 cycles at a rate of 3°C/min. The VPTT was determined from the onset temperature (*T_onset_*) and peak temperature (*T_max_*) of the endothermic phase transition peak. Enthalpy differentiation (*ΔH*) was determined by integration of the peak. Reported values were obtained from the second heating cycle to remove thermal history.

### 4.11 Compressive Mechanical Properties

Hydrogel discs (∼6 mm × ∼1.8 mm, *d* × *t*; *N ≥* 5 from different hydrogel sheets) were blotted with a Kim Wipe and subsequently compressed using an Instron 5944 at RT. A disc was subjected to a 0.2 N preload prior to compression. A strain rate of 1 mm min^−1^ was applied to create a constant compressive force until fracture, determined from a drop in compressive strain (*ε_c_*). The compressive modulus (*E_c_*) was determined using the linear region of the stress–strain curve (0–10% strain). The compressive strength (*σ_c_*) was determined as the stress at failure. Toughness (*U_c_*) was determined from the area under the stress–strain curve using a trapezoidal Reimann sum approximation.

### 4.12 Tensile Mechanical Properties

Tensile properties were determined using an Instron 5944 at RT. Hydrogel samples were prepared using a dog‐bone die (∼3 mm × ∼30 mm, *w* × *gauge length*; *N ≥* 3 from different hydrogel sheets) and blotted with a Kim Wipe. The specimen was then placed in the tensile clamps and subjected to a 0.1 N preload force, followed by a strain rate of 10 mm min^−1^. The tensile modulus (*E_t_*) was obtained from the linear region of the stress–strain curve (0–10% strain). The tensile strength (*σ_f_*) of the hydrogel was defined as the stress at failure. Ultimate tensile strain (*ε_t_*) was defined as the strain at failure. Toughness (*U_t_*) was likewise determined from the area under the stress–strain curve using a trapezoidal Reimann sum approximation.

### 4.13 Lap Shear Strength

The interfacial shear strength of the ‘CAB wall’ (i.e., DN hydrogel) to the ‘CAB cap’ (i.e., *semi‐IPN‐PA/[+]PE*) was determined. A designated DN hydrogel strip (∼40 mm × ∼10 mm, *l* × *w*; *N* ≥ 3, from different hydrogel sheets) and a *semi‐IPN‐PA/[+]PE* hydrogel strip (∼40 mm × ∼10 mm, *l* × *w*; *N* ≥ 3, from different hydrogel sheets) were blotted with a Kim Wipe. The two hydrogel strips were overlapped to form a 10 mm^2^ contact area, and a 500 g weight was placed atop this area for 1 min. The resulting construct was submerged in DI water (ON, RT), and tested the following day with an Instron 5944 at RT using a modified lap shear setup [65]. A length of 1 cm^2^ of each end of the construct was inserted into the tension clamps, with each also containing an aluminum plate (∼70 mm × ∼13 mm × ∼3.15 mm, *l* × *w* × *t*) to prevent rotation during testing. Following application of a preload force (0.1 N), the clamps were displaced at a rate of 100 mm min^−1^ until failure. Interfacial shear strength was defined as the point of failure of either the interface or the hydrogel fracture.

### 4.14 Glucose Diffusion Coefficient

Glucose diffusion studies of a ‘CAB wall’ (i.e., a DN hydrogel) planar sheets were conducted at RT (22°C) and 37°C. Specimens (20 mm × 1.8 mm, *l* × *w*; *N = 3*) were placed in a PermeGear side‐by‐side diffusion chamber. The receiver and donor chambers contained 7 mL of DI water and 7 mL of a designated glucose solution (10 mg/mL), respectively. A constant solution concentration was achieved by stirring the solution in each chamber at 200 rpm, and the solution temperature maintained using a water jacket and pump system. A 50 µL aliquot of solution was collected from each chamber every 10 min over 3 h. The glucose concentration of the collected solution from the receiver chamber was determined using a YSI 2700 Select Biochemistry Analyzer. Fick's laws of diffusion were used to determine the glucose diffusion coefficient (*D)* of the hydrogel per Equation (2) [38, 49, 57]. *Q* is the overall quantity of glucose transferred through the hydrogel at time *t. C_0_* is the initial glucose concentration (10 mg/mL) in the donor chamber, *A* is the surface area of the exposed hydrogel (1.767 cm^2^) during diffusion, and *L* is the thickness of the hydrogel sample (∼1.8 mm). The slope of the linear portion of the “*Q* vs *t”* graphs was used to determine *D*, following any initial lag time.

(2) $$\begin{array}{c} Q=\frac{\mathrm{AD}C_{0}}{L}\times \left(t-\frac{L^{2}}{6D}\right) \end{array}$$

### 4.15 Mesh Size

The approximate mesh size of each ‘CAB wall’ (i.e., a DN hydrogel) was determined through a series of FITC‐dextran (4k, 10k, 20k, and 40k g/mol) diffusion experiments with known hydrodynamic diameters (*D_h_* ∼ 3, 4, 7, and 10 nm, respectively) [82, 83]. A swollen hydrogel disc (∼11 mm × ∼2 mm, *d* × *t*; *N *= 3, from different hydrogel sheets) was soaked in 1 mL of FITC‐dextran solution (0.01 mg/mL in DI water) at RT for 24 h, allowing for diffusion of the dextran into the hydrogel to occur. Following completion of the 24‐h soak, the disc was removed from the FITC‐dextran solution, and the surface of the hydrogel was blotted with a Kim Wipe. The hydrogel disc was then placed in 1 mL of DI for 24 h. A 200 µL aliquot was removed from the supernatant, and its fluorescence intensity was tested using a Biotek Cytation 5 (excitation wavelength (*λ*_ex_) at 480 nm; emission wavelength (*λ*_em_) from 510 to 600 nm). FITC‐dextran diffusion was quantified by converting fluorescence intensity into a calculated concentration via an established calibration curve (fluorescent intensity vs. FITC‐dextran concentration, *R*‐squared value > 0.99) (Figure S3). A calculated concentration of ≤0.00004 mg/mL was considered negligible FITC‐dextran diffusion as this correlated with the average value of DI water.

### 4.16 CAB Construction and FITC‐Dextran Leaching

The CAB was constructed in a dark room to avoid prematurely exciting the FITC fluorophore of FITC‐dextrans. A DN hydrogel hollow rod (∼2.5 mm × ∼5 mm, post‐swelling *OD* × *l*) was blotted with a Kim Wipe to remove surface water and water from the central cavity. The cavity was filled with 1.5 µL of a designated FITC‐dextran solution (0.45 mg/mL). *Semi‐IPN‐PA/[+]PE* discs (∼2.5 mm × ∼1 mm, *d* × *t*; from a single sheet) were used to seal the ends of the rod by applying pressure for 1 min by hand, resulting in a constructed CAB. The resulting CABs (*N* = 3) were each placed into a 0.5 mL microcentrifuge tube that contained 150 µL of DI water and stored at RT. A 50 µL aliquot of the surrounding supernatant was removed at the designated time point: 15 min, 30 min, 45 min, 1 h, 2 h, 4 h, 6 h, 8 h, 1 day, 2 days, 3 days, 4 days, 5 days, 6 days, and 7 days. The fluorescence intensity of the aliquot was determined using a Biotek Cytation 5 (excitation wavelength (*λ*_ex_) at 480 nm; emission wavelength (*λ*_em_) from 510 to 600 nm). The removed aliquot was subsequently transferred back into solution after testing so that the supernatant solution was not depleted. To simulate total FITC‐dextran leaching from the CAB, 1.5 µL of FITC‐dextran solution was injected directly into 150 µL of DI water. A 50 µL aliquot was transferred to a microplate reader, and the total fluorescence intensity was quantified. FITC‐dextran percent lost was determined as shown in Equation (3).

(3) $$\begin{array}{c} FITC-Dextran\;loss\;\left(\%\right)=\frac{\mathrm{Measured}\;\mathrm{fluorescence}\;\mathrm{intensity}}{\mathrm{Total}\;\mathrm{fluorescence}\;\mathrm{intensity}}\times 100 \end{array}$$

### 4.17 Statistical Analysis

Analyses were conducted with GraphPad Prism (version 9.3.1) using analysis of variance (ANOVA) testing. An alpha value of 0.05 was standard across all analyses, and data were reported as statistically significant with *p* < 0.05. All data were reported as mean ± standard deviation.

## Conflicts of Interest

The authors declare no conflicts of interest.

## Supporting information




**Supporting File**: mabi70092‐sup‐0001‐SuppMat.docx.

## Data Availability

Raw data is available via the Texas Data Repository (TDR)
