# Supplementary material for: Advancing the Development of Subcutaneous Glucose Biosensors: Cargo‐Carrying Adhesive Biosensor Systems (CABs)
Source: Macromol Biosci. 2025 Oct 12;26(1):e00445. doi: 10.1002/mabi.202500445 (PMC12829525; doi:10.1002/mabi.202500445)
Supplement: Supplementary file 1 — Supporting File: mabi70092‐sup‐0001‐SuppMat.docx. [file MABI-26-e00445-s001.docx]

Supporting Information

**Advancing the Development of Subcutaneous Glucose Biosensors: Cargo-Carrying Adhesive Biosensor Systems (CABs)**

Carolina I. Martinez, Theodore S. Ferrell, Varshitha M. Krishnan, and Melissa A. Grunlan*

C. I. Martinez

Department of Chemistry

Texas A&M University, College Station, TX 77843-3003 (USA)

T.S. Ferrell, V. M. Krishnan

Department of Biomedical Engineering

Texas A&M University, College Station, TX 77843-3003 (USA)

Prof. M. A. Grunlan

Department of Biomedical Engineering, Department of Materials Science & Engineering, Department of Chemistry, Center for Remote Health Technologies Systems

Texas A&M University, College Station, TX 77843-3003 (USA)

Email: [mgrunlan@tamu.edu](mailto:mgrunlan@tamu.edu)


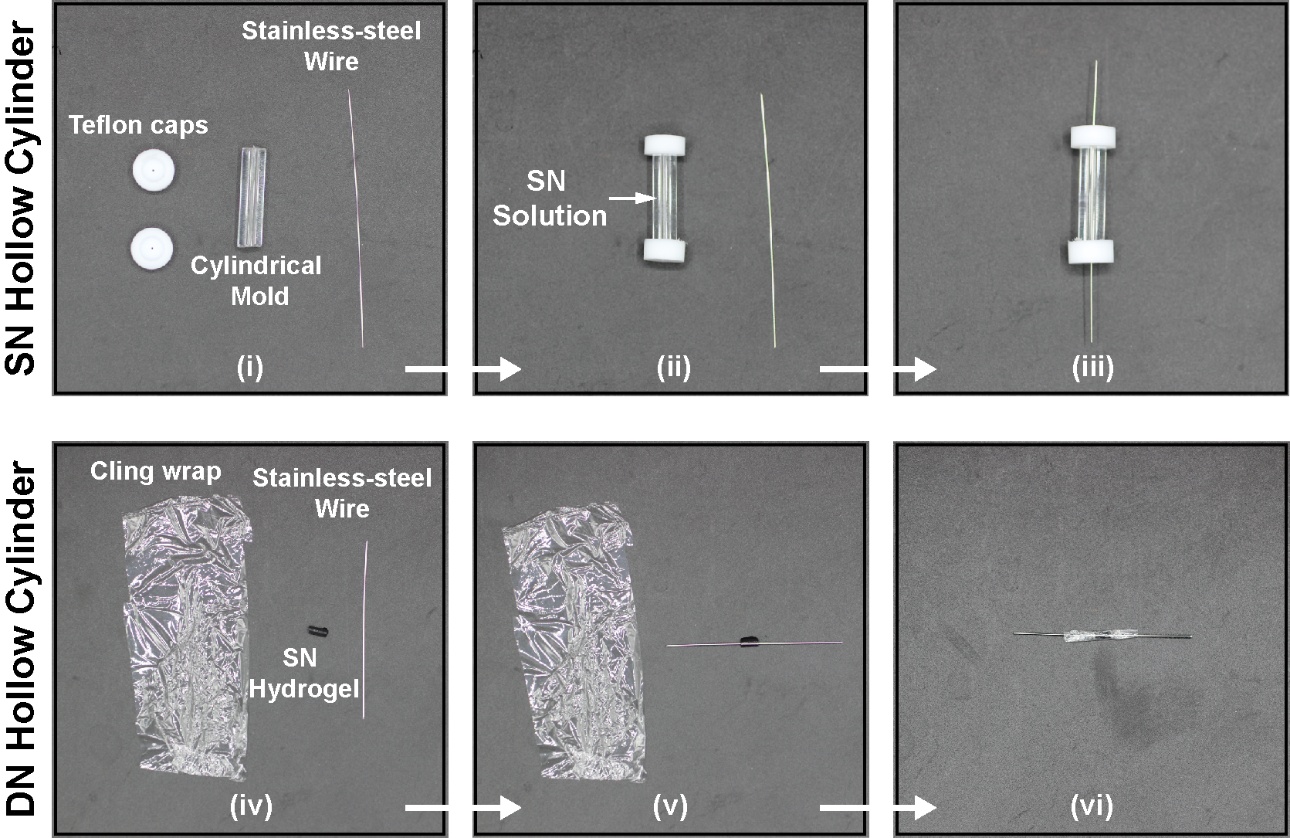


**Figure S1.** Double network (DN) hollow cylinder fabrication via a 2 step UV curing process. **Top row:** A single network (SN) precursor solution is injected in the central cavity of a cylindrical glass mold and sealed with Parafilm^®^. Teflon coated caps are placed on the ends and a stainless-steel wire is inserted through the caps to form a hollow central cavity. **Bottom row:**  A stainless-steel wire is inserted through the central cavity of the SN hydrogel and tightly wrapped in cling wrap.

**Table S1**. VPTT values.

| **Composition** | **T_onset_ [°C]** | **T_max_ [°C]** | **ΔH [J/g]** |
| --- | --- | --- | --- |
| *G_1_ ^a^* | 38.04 ± 0.78 | 41.90 ± 1.55 | 2.8 ± 0.5 |
| *G_2_ ^a^* | 37.13 ± 0.56 | 41.40 ± 0.21 | 2.0 ± 0.4 |
|  |  |  |  |
| *G_3_* | 30.89 ± 0.22 | 33.52 ± 0.08 | 2.8 ± 0.3 |
| *G_3_-AMPS_5_* | 31.91 ± 0.11 | 34.29 ± 0.13 | 2.1 ± 0.2 |
| *G_3_-AMPS_10_* | 31.88 ± 0.08 | 34.45 ± 0.03 | 2.4 ± 0.0 |
| *G_3_-AMPS_25_* | 33.44 ± 0.10 | 36.79 ± 0.07 | 2.1 ± 0.1 |
| *G_3_-AMPS_50_* | 36.01 ± 0.21 | 40.91 ± 0.11 | 1.3 ± 0.2 |
| *G_3_-AMPS_60_* | 37.09 ± 0.06 | 42.66 ± 0.22 | 1.3 ± 0.2 |
| *G_3_-AMPS_70_* | 37.86 ± 0.08 | 43.99 ± 0.12 | 1.1 ± 0.1 |
| *G_3_-AMPS_80_* | 38.24 ± 1.17 | 45.54 ± 0.35 | 0.9 ± 0.2 |
| *G_3_-AMPS_90_* | 40.92 ± 0.37 | 47.96 ± 0.23 | 0.6 ± 0.1 |
| *G_3_-AMPS_100_ ^b^* | - | - | - |
|  |  |  |  |
| *G_4_* | 35.79 ± 0.36 | 41.57 ± 0.03 | 2.2 ± 0.2 |
| *G_4_-AMPS_60_* | 37.27 ± 0.04 | 42.14 ± 0.16 | 1.5 ± 0.1 |
| *G_4_-AMPS_70_* | 38.75 ± 0.30 | 44.62 ± 0.18 | 0.9 ± 0.03 |
| *G_4_-AMPS_80_* | 39.76 ± 0.41 | 45.51 ± 0.05 | 0.7 ± 0.04 |

(a): Data previously reported^[1]^

(b): An endothermic peak was not present in the differential thermogram.

**Table S2.** Equilibrium water content (EWC).

| **Composition** | **EWC**  **[%]** |  |
| --- | --- | --- |
| *G_1_* | 88.60 ± 0.32 | |
| *G_2_* | 88.95 ± 0.30 | |
|  |  | |
| *G_3_* | 91.34 ± 0.37 | |
| *G_3_-AMPS_50_* | 90.66 ± 0.76 | |
| *G_3_-AMPS_60_* | 90.70 ± 0.60 | |
| *G_3_-AMPS_70_* | 91.68 ± 0.15 | |
| *G_3_-AMPS_80_* | 93.96 ± 3.51 | |
| *G_3_-AMPS_90_* | 92.62 ± 0.35 | |
| *G_3_-AMPS_100_* | 91.57 ± 0.38 | |
|  |  | |
| *G_4_* | 87.52 ± 0.20 | |
| *G_4_-AMPS_60_* | 89.83 ± 0.29 | |
| *G_4_-AMPS_70_* | 90.54 ± 0.20 | |
| *G_4_-AMPS_80_* | 90.48 ± 0.28 | |
| *semi-IPN-PA/[+]PE* | 48.51 ± 0.58 | |

**Table S3.** Hydrogel compressive mechanical properties.

| **Composition** | **Modulus (*E_c_*)**  **[MPa]** | **Strength (σ_c_)**  **[MPa]** | **Toughness (U_c_)**  **[MJ m^-3^]** | **Ultimate Strain (*ε_c_*)**  **[%]** |
| --- | --- | --- | --- | --- |
| *PEG* | 0.36 ± 0.03 | 1.65 ± 1.06 | 0.22 ± 0.11 | 61.77 ± 6.99 |
|  |  |  |  |  |
| *G_1_* | 0.93 ± 0.05 | 2.03 ± 0.30 | 0.19 ± 0.03 | 38.59 ± 1.85 |
| *G_2_* | 0.74 ± 0.05 | 1.12 ± 0.18 | 0.12 ± 0.02 | 37.11 ± 1.25 |
|  |  |  |  |  |
| *G_3_* | 0.76 ± 0.09 | 2.40 ± 0.25 | 0.28 ± 0.04 | 45.37 ± 4.07 |
| *G_3_-AMPS_50_* | 0.75 ± 0.04 | 1.78 ± 0.14 | 0.21 ± 0.02 | 43.36 ± 1.39 |
| *G_3_-AMPS_60_* | 0.88 ± 0.03 | 2.20 ± 0.19 | 0.24 ± 0.03 | 41.70 ± 1.27 |
| *G_3_-AMPS_70_* | 0.54 ± 0.03 | 1.98 ± 0.16 | 0.23 ± 0.2 | 46.63 ± 1.19 |
| *G_3_-AMPS_80_* | 0.53 ± 0.02 | 1.89 ± 0.17 | 0.21 ± 0.03 | 45.81 ± 1.27 |
| *G_3_-AMPS_90_* | 0.61 ± 0.02 | 2.42 ± 0.30 | 0.32 ± 0.06 | 50.80 ± 2.12 |
| *G_3_-AMPS_100_* | 0.84 ± 0.02 | 1.61 ± 0.13 | 0.17 ± 0.20 | 38.90 ± 1.16 |
|  |  |  |  |  |
| *G_4_* | 0.68 ± 0.08 | 3.28 ± 0.11 | 0.45 ± 0.04 | 54.85 ± 2.21 |
| *G_4_-AMPS_60_* | 0.86 ± 0.05 | 3.36 ± 0.26 | 0.48 ± 0.06 | 51.67 ± 1.83 |
| *G_4_-AMPS_70_* | 0.84 ± 0.05 | 3.38 ± 0.29 | 0.48 ± 0.08 | 50.84 ± 3.39 |
| *G_4_-AMPS_80_* | 0.91 ± 0.07 | 3.28 ± 0.72 | 0.45 ± 0.13 | 49.53 ± 3.46 |
| *semi-IPN-PA/[+]PE****^a^*** | 0.25 ± 0.07 | 25.99 ± 1.95 | 2.69 ± 0.34 | 85 ± 0.0 |

**(a**) A constant compressive strain was applied until 85% strain

**Table S4.** Hydrogel tensile mechanical properties

| **Composition** | **Tensile Modulus (*E_t_*)**  **[kPa]** | **Tensile Strength (σ_t_)**  **[kPa]** | **Toughness  (U_t_)**  **[kJ m^-3^]** | **Ultimate Strain** **(*ε_t_*)**  **[%]** |
| --- | --- | --- | --- | --- |
| *G_3_* | 430.4 ± 20.2 | 84.36 ± 8.01 | 7.35 ± 1.60 | 15.86 ± 1.95 |
| *G_3_-AMPS_50_* | 617.5 ± 26.6 | 116.50 ± 16.65 | 8.92 ± 2.03 | 15.09 ± 1.59 |
| *G_3_-AMPS_60_* | 572.0 ± 36.6 | 97.06 ± 15.58 | 7.09 ± 1.74 | 14.87 ± 1.85 |
| *G_3_-AMPS_70_* | 546.6 ± 39.4 | 109.90 ± 21.60 | 9.64 ± 3.52 | 16.44 ± 3.33 |
| *G_3_-AMPS_80_* | 536.4 ± 17.7 | 124.70 ± 7.60 | 12.09 ± 1.14 | 18.84 ± 0.97 |
| *G_3_-AMPS_90_* | 586.1 ± 19.8 | 147.00 ± 18.08 | 16.11 ± 3.19 | 21.14 ± 2.26 |
| *G_3_-AMPS_100_* | 619.9 ± 48.7 | 151.70 ± 19.14 | 14.13 ± 3.01 | 18.83 ± 2.26 |
|  |  |  |  |  |
| *G_4_* | 602.3 ± 34.0 | 333.70 ±28.75 | 42.17 ± 4.51 | 42.17 ± 4.51 |
| *G_4_-AMPS_60_* | 736.8 ± 50.4 | 195.50 ± 10.59 | 19.76 ± 0.89 | 20.96 ± 0.86 |
| *G_4_-AMPS_70_* | 701.8 ± 3.3 | 601.10 ± 62.02 | 87.55 ± 14.42 | 37.98 ± 2.60 |
| *G_4_-AMPS_80_* | 693.9 ± 43.1 | 260.50 ± 84.93 | 29.28 ± 11.97 | 24.60 ± 3.93 |
| *semi-IPN-PA/[+]PE* | 66.3 ± 8.7 | 461.4 ± 157.6 | 697.6 ± 178.1 | 550.9 ± 39.94 |


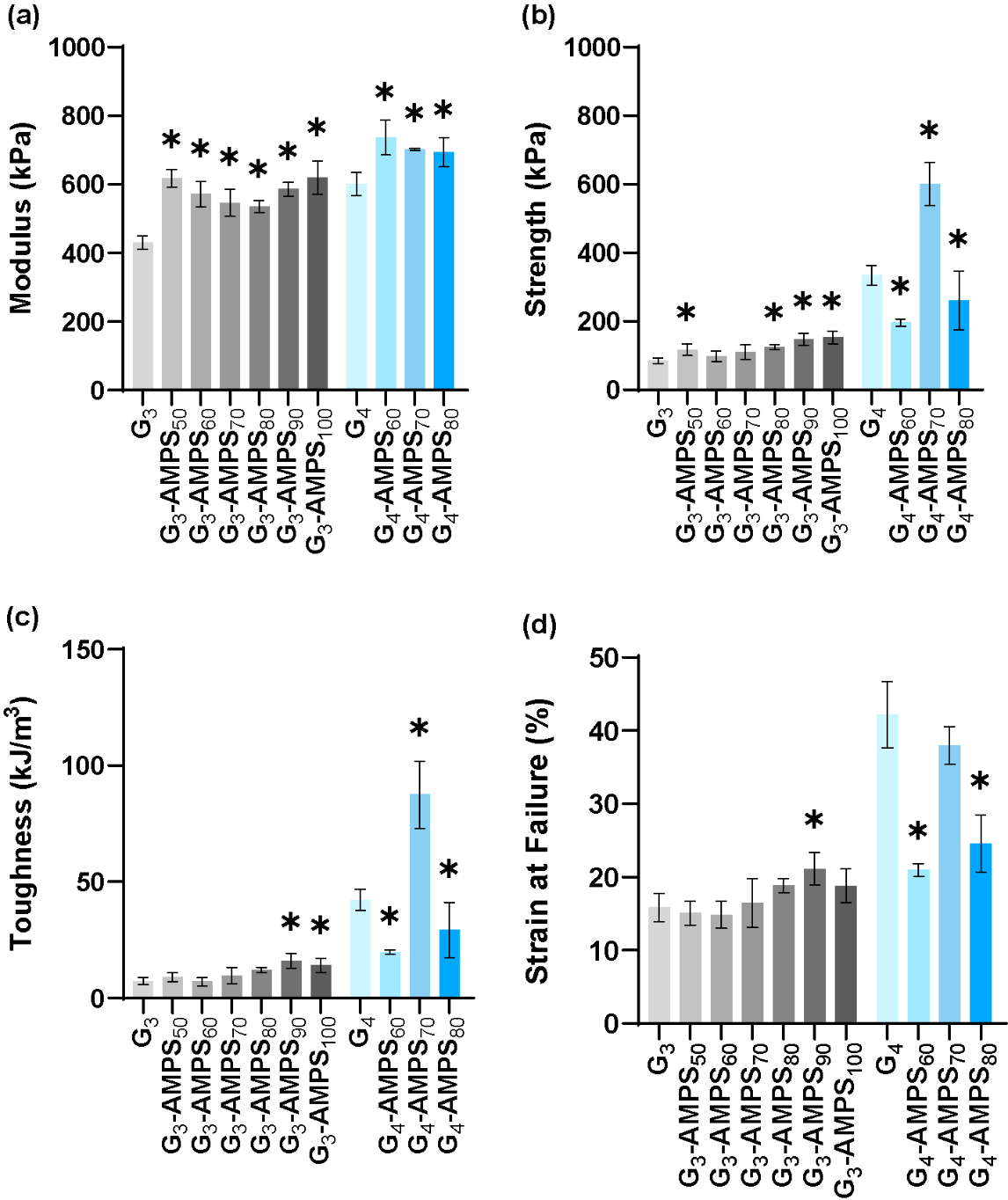


**Figure S2.** Tensile properties of hydrogels. *p < 0.05 versus composition without [-] AMPS in the 2^nd^ network (i.e., G_3_ or G_4_).


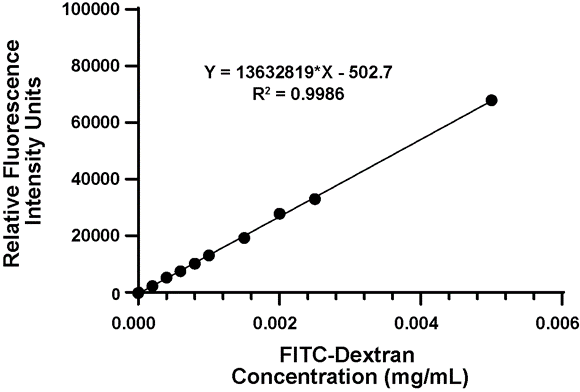


**Figure S3.** Sample FITC-dextran (4k g/mol) calibration curve

**Table S5.** Glucose diffusion coefficient (D) values at room temperature (22 °C).

| **Composition** | **Glucose Diffusion Coefficient**  **[x10^-6^ cm^2^s^-1^]** |
| --- | --- |
| *PEG* | 2.73 ± 0.10 |
| *G_2_* | 2.26 ± 0.02 |
| *G_3_* | 2.50 ± 0.08 |
| *G_3_-AMPS_50_* | 2.62 ± 0.27 |
| *G_3_-AMPS_60_* | 2.46 ± 0.17 |
| *G_3_-AMPS_70_* | 2.45 ± 0.08 |
| *G_3_-AMPS_80_* | 2.56 ± 0.07 |
| *G_3_-AMPS_90_* | 2.53 ± 0.10 |
| *G_3_-AMPS_100_* | 2.45 ± 0.20 |
| *G_4_* | 1.96 ± 0.14 |
| *G_4_-AMPS_60_* | 2.51 ± 0.18 |
| *G_4_-AMPS_70_* | 2.20 ± 0.13 |
| *G_4_-AMPS_80_* | 2.47 ± 0.07 |

**Table S6.** Glucose diffusion coefficient (D) at 37 °C.

| **Composition** | **Glucose Diffusion Coefficient**  **[x10^-6^ cm^2^s^-1^]** |
| --- | --- |
| *PEG* | 3.37 ± 0.05 |
| *G_3_-AMPS_70_* | 3.56 ± 0.17 |

**Table S5.** Lap shear strength between DN hydrogels (i.e., CAB wall) and semi-IPN-PA/[+]PE (i.e., CAB cap). Maximum weight (g) withstood by construct per **Figure S4.**

| **Composition** | **Shear Strength**  **[kPa]** | **Max Weight**  **[g]** |
| --- | --- | --- |
| *G_3_* | - | - |
| *G_3_-AMPS_50_* | 4.19 ± 0.34 | 30.0 |
| *G_3_-AMPS_60_* | 4.15 ± 0.34 | 30.0 |
| *G_3_-AMPS_70_* | 5.38 ± 0.36 | 50.0 |
| *G_3_-AMPS_80_* | 5.11 ± 0.14 | 40.0 |
| *G_3_-AMPS_90_* | 5.89 ± 0.47 | 60.0 |
| *G_3_-AMPS_100_* | 7.12 ± 0.17 | 70.0 |
|  |  |  |
| *G_4_* | - | - |
| *G_4_-AMPS_60_* | 6.47 ± 0.86 | 100.0 |
| *G_4_-AMPS_70_* | 5.47 ± 0.40 | 110.0 |
| *G_4_-AMPS_80_* | 6.55 ± 0.46 | 90.0 |


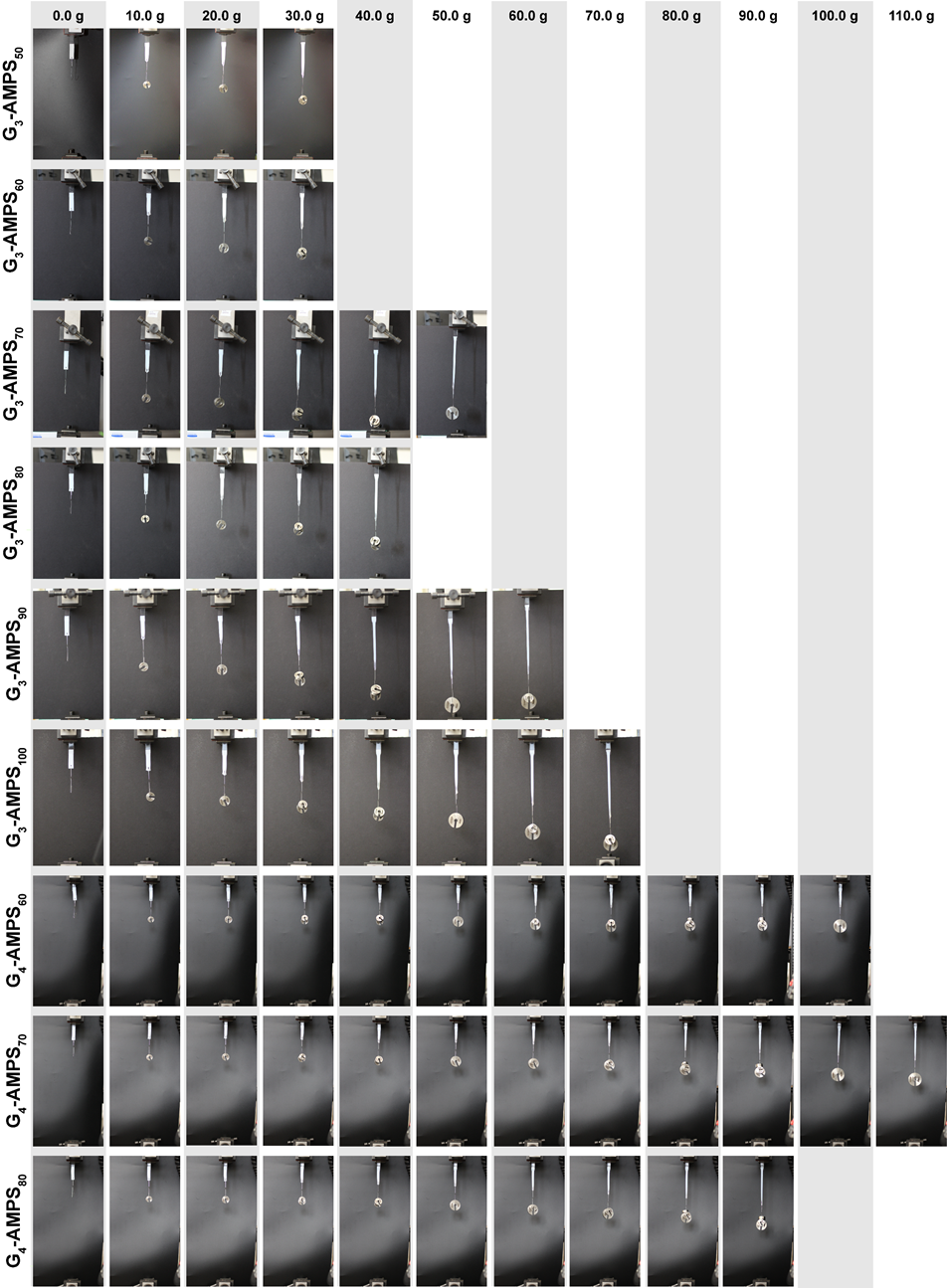


**Figure S4.** A photo series displaying the masses that can be placed on adhered hydrogel constructs: [-] anionic DN hydrogels (top, clear) adhered to [+] cationic *sIPN-PDADMAC* hydrogel (bottom, opaque).

**Supporting Information Citation**

[1] Dong, P.; Schott, B. J.; Means, A. K.; Grunlan, M. A. Comb architecture to control the selective diffusivity of a double network hydrogel. *ACS Appl. Polym. Mater.* **2020**, *2*, 5269-5277. DOI: 10.1021/acsapm.0c00987
